# Relevant Developments in the Use of Three‐Component Reactions for the Total Synthesis of Natural Products. The last 15 Years

**DOI:** 10.1002/open.202300306

**Published:** 2024-04-22

**Authors:** Enrique L. Larghi, Andrea B. J. Bracca, Sebastian O. Simonetti, Teodoro S. Kaufman

**Affiliations:** ^1^ Instituto de Química Rosario IQUIR (CONICET-UNR) Facultad de Ciencias Bioquímicas y Farmacéuticas Universidad Nacional de Rosario (UNR) Suipacha 531 2000 Rosario Argentina

**Keywords:** Heterocycles, Multicomponent reactions, Natural products, Step-economy and efficiency, Total synthesis

## Abstract

Multicomponent reactions (MCRs) offer a highly useful and valuable strategy that can fulfill an important role in synthesizing complex polysubstituted compounds, by simplifying otherwise long sequences and increasing their efficiency. The total synthesis of selected natural products employing three‐component reactions as their common strategic MCR approach, is reviewed on a case‐by‐case basis with selected targets conquered during the last 15 years. The revision includes detailed descriptions of the selected successful sequences; relevant information on the isolation, and bioactivity of the different natural targets is also briefly provided.

## Graphical abstract

Multicomponent reactions (MCRs) are a valuable strategy toward complex polysubstituted compounds because they simplify long sequences, increasing their efficiency. The total synthesis of natural products achieved since 2004 through the use of three‐component reactions as a strategic MCR approach is reviewed. Successful sequences are described, along with relevant information on the isolation and bioactivity of the selected targets.

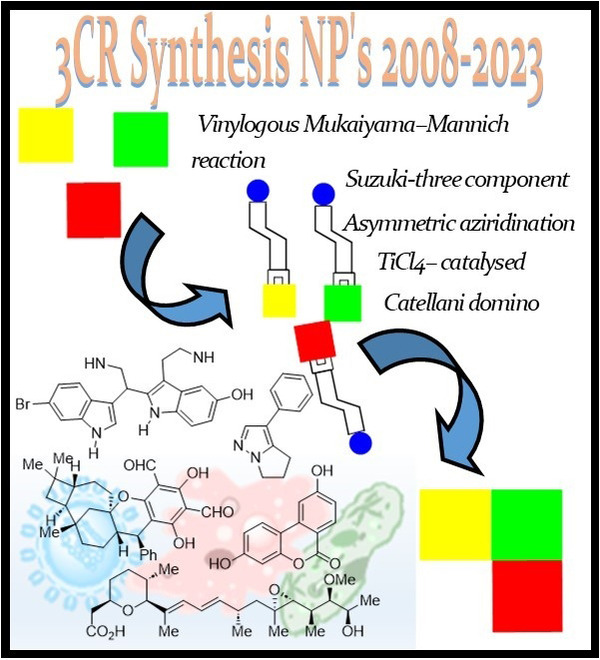

## 1 Introduction

Multicomponent reactions (MCRs) are an eclectic group of chemical transformations, where each one of these reactions embodies an efficient and powerful synthetic strategy for the step‐abbreviated construction of complex molecules from rather simple starting materials. In an MCR, three or more reactants undergo concomitant, simultaneous, or sequential (tandem, domino, cascade) participation in a “one pot” chemical transformation, without changing the solvent. This results in the formation of a structurally more complex product in this single step.^[1]^ Three‐component reactions (3CRs) are the simplest scenarios for carrying out MCRs.

In general, traditional synthetic approaches involve numerous sequential reactions, which result in prolonged reaction times and increased chances of undesired side reactions. In contrast, MCRs reduce the number of reaction steps required, allowing for the simultaneous incorporation of multiple building blocks, streamlining and shortening the synthetic sequence. This efficiency gain is crucial for complex molecules with intricate structures, such as natural products, where MCRs can ease their synthesis by providing notoriously abbreviated and time‐saving routes.

The convergent nature of MCRs allows for the modular assembly of intermediate fragments, which can be subsequently joined together to form the final complex target molecule. This modular approach facilitates the optimization of individual fragments and provides flexibility in the overall synthetic strategy. Additionally, MCRs often exhibit a high atom economy, minimizing waste production and also promoting sustainability in synthetic chemistry.

No less important is their wide scope and compatibility with an ample range of functional groups and reaction conditions.

A high number of MCRs have been successfully employed in diverse chemical environments, including aqueous media, and under mild reaction conditions. This versatility allows for the incorporation of sensitive functional groups at the early stages of a complex synthesis. This is highly relevant in the field of natural product synthesis because they usually have multiple functional groups and stereocenters.

In this context of challenge towards simplicity and efficiency, MCRs have attracted considerable attention in recent years, and they have become a central topic in organic chemistry research.

This article aims to update previous reviews that covered part of the field^[2]^ and to complement our recent publication on the use of Ugi and related MCRs for natural product synthesis.^[3]^ Hence, the focus is placed on applying 3CR to the total syntheses of selected natural products reported in the last 15 years, emphasizing their synthetic relevance. Despite the structural diversity of the targets, they have been ordered considering the presence of heteroatoms (N, O) and the complexity of their heterocyclic rings. For the sake of readability, brief details on the isolation, previous syntheses, and spectrum of bioactivity precede the discussion of the synthesis of each selected natural product.

## 2 Synthetic Endeavors Related to Natural Products

**Figure 1:**
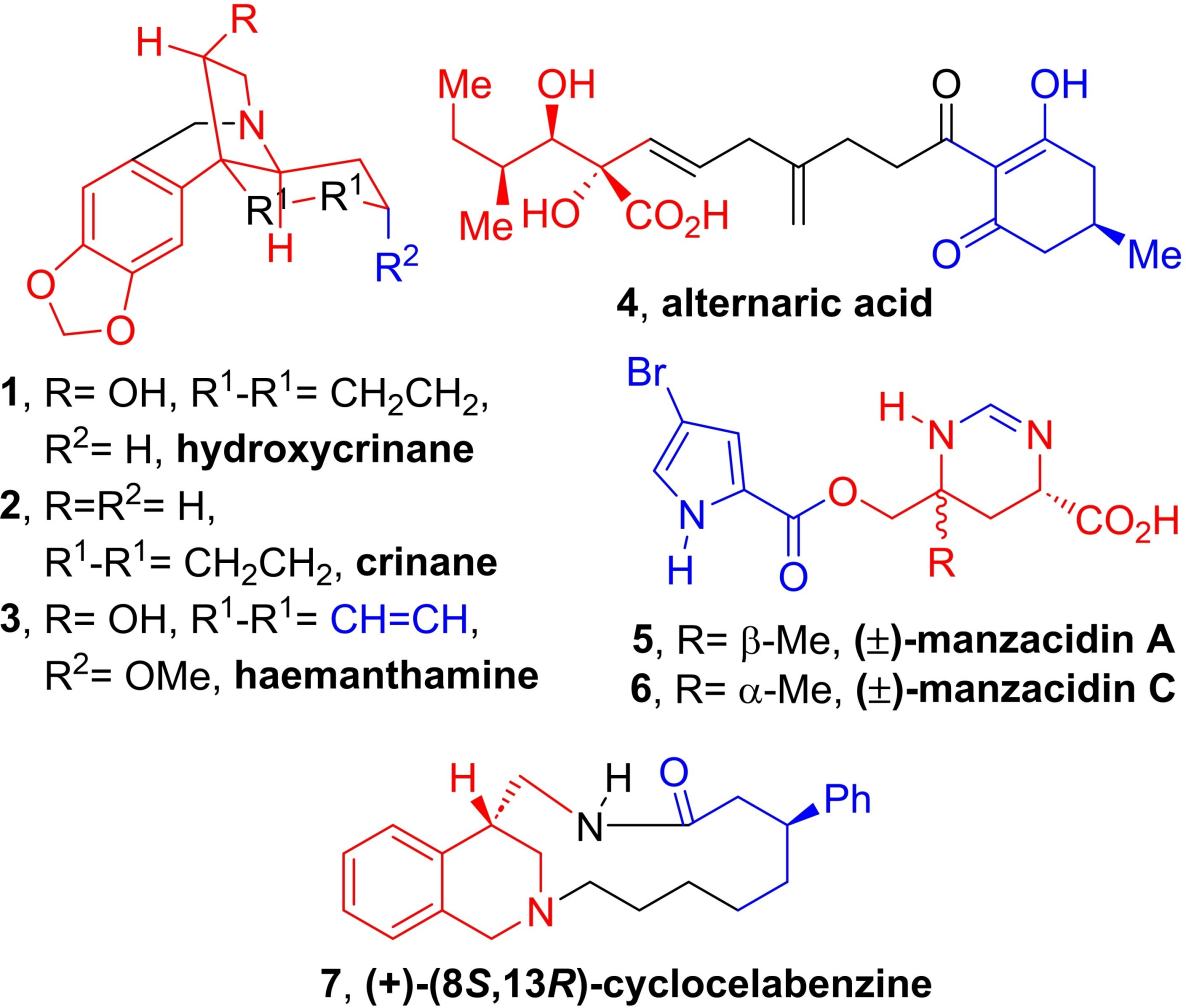
Selection of natural products recently targeted for partial or formal syntheses employing MCRs. In black and red, the final molecular fragments that included the MCR‐based efforts; in red the molecular fragments prepared with the aid of MCRs.

The objective of this review is to cover the recently disclosed total syntheses of natural products achieved by the use of 3CRs. However, it should also be taken into account that aside from the total synthesis of natural products in themselves, several formal total syntheses of natural products employing MCRs have been recently reported (Figure 1).

This includes, for example, hydroxycrinane (**1**, dihydrodemethoxyhaemanthamine), which may set the stage for the access to crinane (**2**) and hemanthamine‐ (**3**) type Amarillidaceae alkaloids,^[4]^ alternaric acid (**4**),^[5]^ (±)‐manzacidin A and C (**5** and **6**),^[6]^ and (+)‐(8*S*,13*R*)‐cyclocelabenzine (**7**).^[7]^ In addition, critical parts of natural products, such as the central core of halichonadins K and L^[8]^ have also been prepared by the use of such a strategy.

Furthermore, MCRs have been used as key steps in the elaboration of natural product analogs and of natural product‐inspired structures, like in the sequential five‐components gram‐scale approach to the rare hexahydropyrrolo[3,2‐*c*]quinoline motif,^[9]^ found in some of the bioactive alkaloids isolated from *Martinella iquitosensis*.

Natural‐product‐like hybrids have also been accessed using MCRs, coupled with other strategies.^[10]^ For example, an organocatalytic/MCR approach resulted in the highly stereoselective synthesis of many new compounds, where the organocatalysts provide the stereocontrol and the MCR contributes its diversity‐generating characteristics. Thus peptidomimetic compounds integrating heterocyclic, lipidic, and sugar moieties have been easily obtained.^[11]^ The diversification of other families of natural products has also been reported.^[12]^

## 3 3CRs‐Based Total Synthesis of Natural Products

### 3.1 Nitrogen‐Containing Natural Products

Discussed in this section are the 3CRs‐based total syntheses of natural products carrying nitrogen. It contains linear amines, such as sphinganine and safingol, as well as heterocycles featuring indolizidine, pyrazole, and pyrrole motifs, including indole alkaloids among the latter. The applied strategies involve asymmetric organocatalysis, the use of titanium complexes as well as catalytic organometallic approaches.

#### 3.1.1 Sphinganine and Safingol

The sphingolipids are a family of compounds involved in cell structure and regulation, including proliferation, differentiation, adhesion, signal transduction, and neuronal repair.^[13]^ Errors in their metabolism result in several diseases, including diabetes, cancers, Alzheimer's disease, heart disease, and neurological disorders.^[14]^

Sphinganine (**8**) is one of the three major core units found in sphingolipids. The natural configuration of sphinganine is the D‐*erythro*; however, it has been observed that the stereochemistry of these compounds can play a large role in their bioactivity. For example, the L‐*threo* diastereomer (safingol, **9**) displays antineoplastic and antipsoriatic activity and has been studied as a protein kinase C inhibitor.^[15]^

**Scheme 1:**
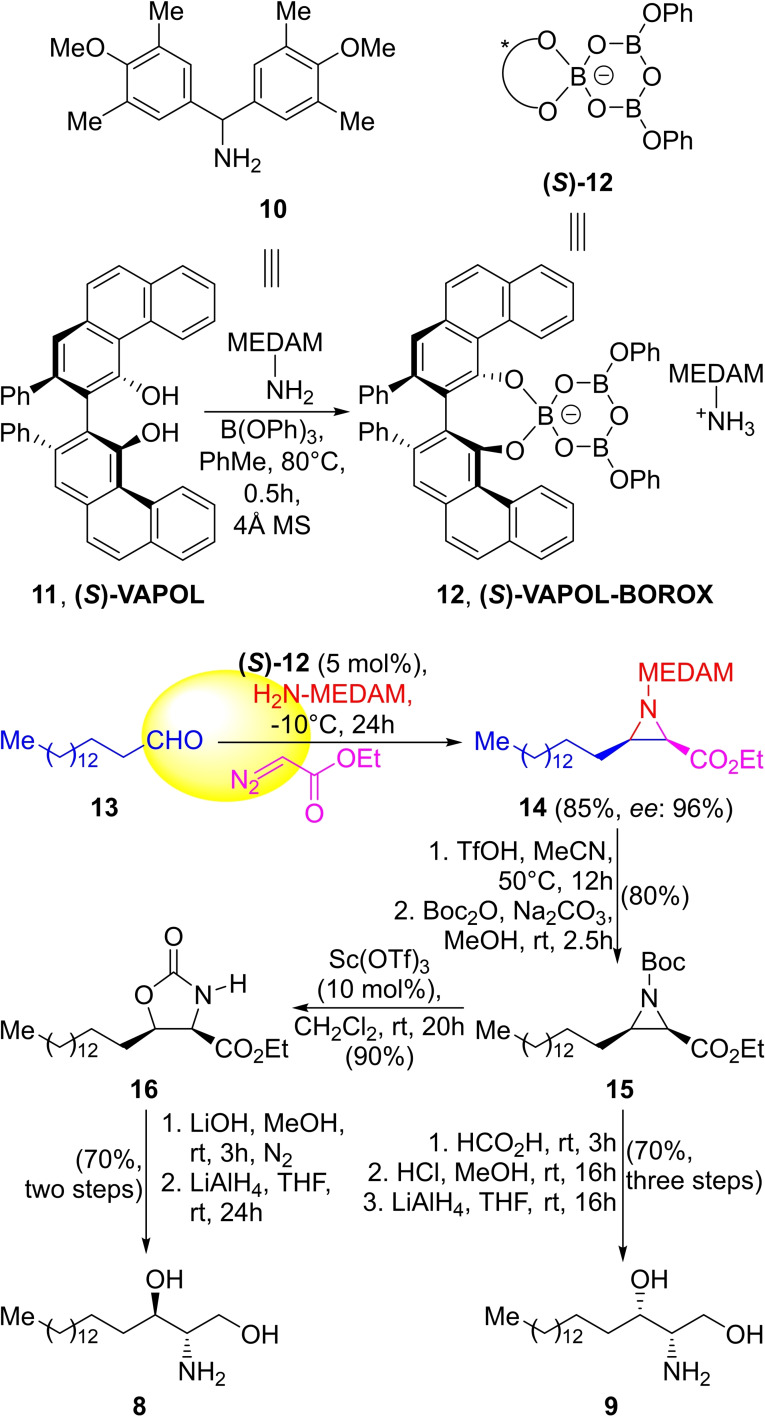
Total syntheses of sphinganine (**8**) and safingol (**9**).

Mukherjee *et al*. developed a three‐component catalytic asymmetric aziridination reaction that involves an aldehyde, an amine, and a diazo compound. A chiral polyborate anion [*(S)‐*VAPOL‐BOROX, **12**], that is assembled *in situ* from the chiral biaryl ligand *S*‐VAPOL (**11**) and B(OPh)_3_ is used as a catalyst, in the presence of an amine [usually tetra‐methyldianisylmethyl (MEDAM) amine, H_2_N‐MEDAM, **10**].^[16]^ In 2014, the same group reported the application of this 3CR to the total syntheses of sphinganine, safingol, and their enantiomers (Scheme 1).^[17]^

The 3CR between the long‐chain aldehyde **13**, ethyl diazoacetate, and **10** catalyzed by **12** afforded the key aziridine **14** in 85 % yield and 96 % *ee*. One of the advantages of this reaction is that it can be run efficiently at a multigram scale.

Deprotection of the MEDAM moiety was carried out with TfOH, and this was followed by preparation of the corresponding Boc‐protected derivative **15** by reaction with Boc_2_O in 80 % overall yield. However, it was noticed that the ring opening of aziridines with oxygen nucleophiles with inversion of configuration requires an electron‐withdrawing group on the nitrogen atom.^[18]^

Therefore, **15** was exposed to neat formic acid at room temperature, which resulted in ring opening with formate anion and subsequent *O*‐ to *N*‐formyl migration. The *N*‐formyl moiety was hydrolyzed with HCl, and finally, the ester was reduced with LiAlH_4_, affording safingol (**9**) in 70 % yield. On the other hand, in order to obtain the diastereomeric **8**, retention of configuration was required after the ring‐opening of the aziridine **15** with an oxygen nucleophile. This was materialized through an Sc(OTf)_3_‐mediated ring expansion strategy to the related oxazolidinone **16**, which was obtained in 90 % yield.^[19]^ Ring opening of the oxazolidinone with LiOH in MeOH and reduction of the ester gave sphinganine (**8**) in 70 % yield.

Alternate routes aiming to carry out the direct ring opening of the unactivated MEDAM aziridines with TFA and removal of the MEDAM group by catalytic hydrogenolysis with Pd(OH)_2_/C have also been studied. The synthesis of the remaining pair of enantiomers was carried out analogously and proceeded in similar yields when the enantiomer of the BOROX catalyst was employed.

**Scheme 2:**
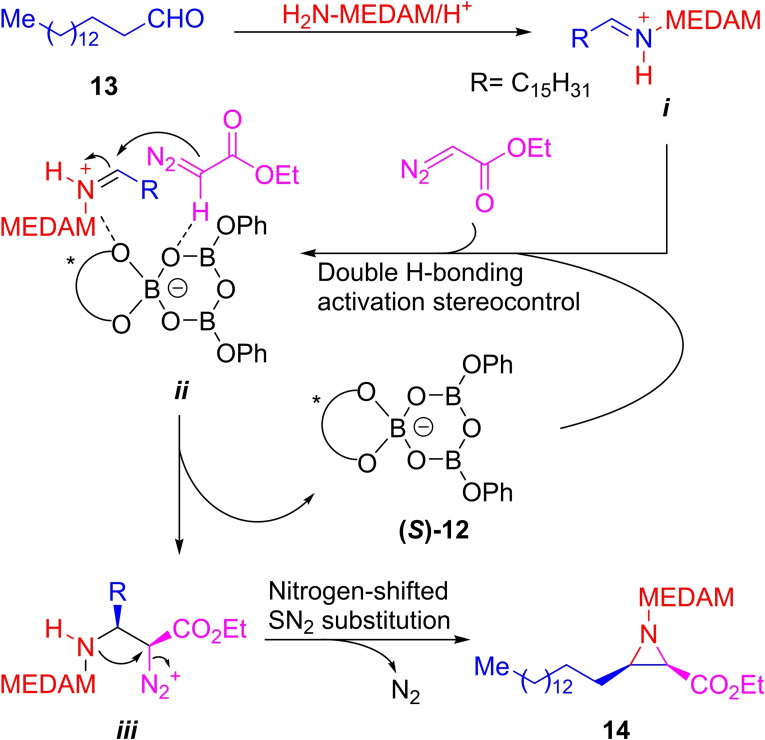
Mechanism to the 3CR asymmetric synthesis of aziridines.

The mechanism of the asymmetric organocatalytic 3CR synthesis of aziridines was studied both theoretically and experimentally.^[20]^ It was suggested that the sequence is initiated by the formation of the protonated intermediate imine ***i*** by the interaction between the aldehyde **13** and MEDAM‐NH_2_, followed by the protonation of the resulting imine (Scheme 2).

Next, a double hydrogen bond interaction between the VAPOL‐BOROX chiral catalyst [**(*S*)‐12**] and both, intermediate ***i*** and ethyl diazoacetate (***ii***), where the *cis* conformation is preferred, would provide activation and stereocontrol, helping to establish the stereochemistry of the product. Interestingly, one of the crucial interactions is the non‐classical hydrogen bond between the Cα proton of the diazo compound with a boroxine oxygen.

Activation of the imine carbon atom would enable its attack by the diazonium carbon with concomitant asymmetric C−C bond formation (***iii***) and release of the organocatalyst. Finally, the intermediate ***iii*** would form the aziridine framework via a nitrogen‐shifted SN_2_ substitution to provide the product **14**.

#### 3.1.2 (+)‐Indolizidine‐Based Alkaloids from Frog Skin

Indolizidine‐based alkaloids (IBAs) seem to be produced by ants, mites, and other arthropods, and accumulated in the skin glands of poison frogs for defense purposes, from where they have been isolated in minute amounts. To date, over 240 different members of this family have been identified.^[21]^

Some IBAs are noncompetitive blockers of the nicotinic receptor channels;^[22]^ hence, potentially useful to inspire drugs against schizophrenia, epilepsy, Parkinson and Alzheimer's disease.^[23]^

Most IBAs have been characterized only by GC‐MS and their stereochemical features are uncertain; furthermore, the absolute configuration of most IBAs remains unknown to date. Therefore, their total synthesis not only proves the power of imagination and the efficiency of new stereoselective methods; they also provide material for their proper stereochemical assignment and for biological testing.

In 2012, the group of Schneider developed a three‐component strategy toward the total synthesis of various substituted IBAs, which relied on a Brønsted acid catalyzed, highly enantioselective vinylogous Mukaiyama–Mannich reaction^[24]^ between an acyclic silyl dienolate, a γ‐oxoester, and *para*‐anisidine to furnish γ‐lactams as key intermediates.^[25]^

**Scheme 3:**
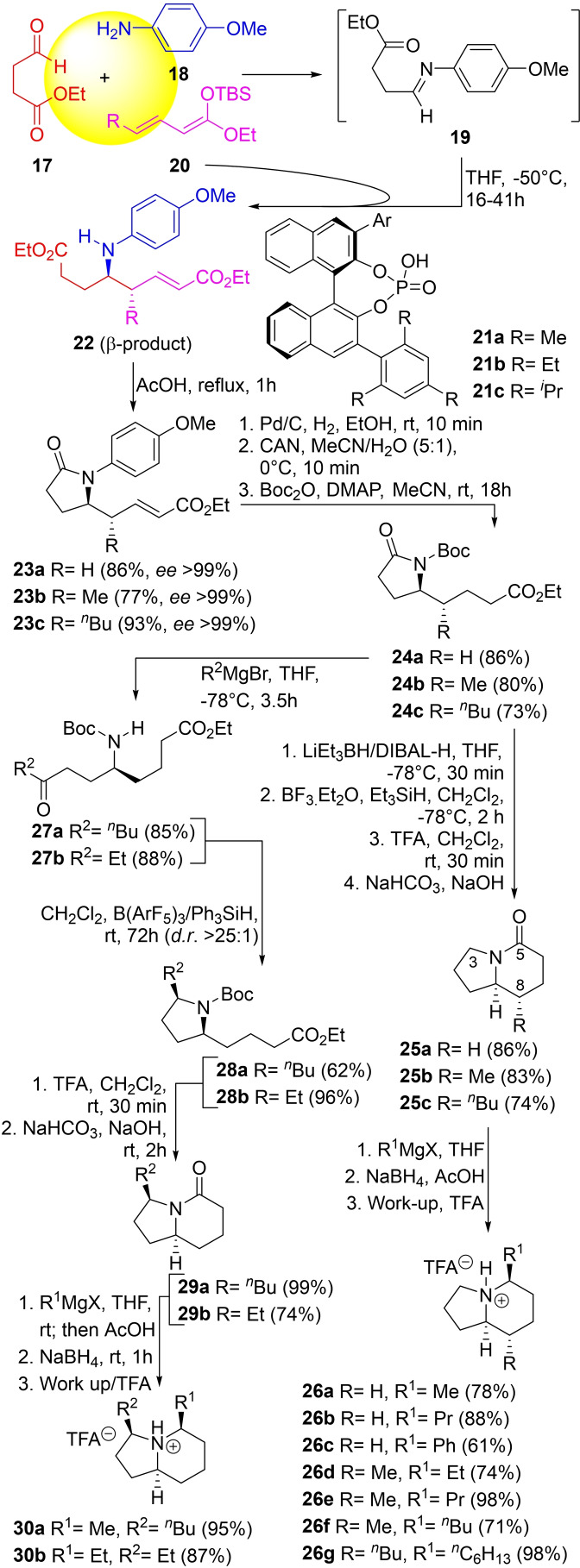
Total syntheses of some naturally occurring indolizidines.

This strategy, which resorted to the use of a few common versatile central building blocks that were made available on a multigram scale, enabled the enantioselective total synthesis of sixteen IBAs isolated from the skin of poisonous frogs, many of them for the first time (Scheme 3).

In the synthetic sequence, aldehyde **17** was made to react with *p*‐anisidine (**18**) in THF at −50 °C, furnishing the imine intermediate **19**, which in turn underwent a diastereoselective addition with the silylketene acetal **20** and resulted in the β‐product type adducts **22**.

The reactions were catalyzed by chiral BINOL‐based^[26]^ phosphoric acid derivatives (**21 a**–**c**),^[27]^ which were chosen to provide only the β‐products, in high yields and enantiomeric excesses.

Exposure of the adducts to refluxing AcOH furnished the pyrrolidones **23 a**–**c** (77–93 % yield), which after double bond hydrogenation and change of the *N*‐protecting group (PMP→Boc) afforded the intermediates **24 a**–**c** in 73–86 % yield.

Compounds **24 a**–**c** served as common intermediates for the preparation of the 3‐ and 8‐mono‐ and 3,8‐di‐substituted compounds **25 a**–**c** through a sequence that entailed deoxygenation of the pyrrolidone through successive reductions, first to the hemiaminal, with DIBAL−H or LiEt_3_BH and then to the pyrrolidine through a cationic reduction with BF_3_.Et_2_O and Et_3_SiH,^[28]^ a TFA‐assisted cyclization to deliver the heterocycle‐derived salts and final freeing of the base. The whole process took place in 74–86 % overall yield.^[29]^

Introduction of the C‐5 substituent was performed in 61–98 % yield through a three‐step sequence, which entailed Grignard addition to the lactam, followed by NaBH_4_‐mediated deoxygenation of the resulting aminal in AcOH and final trifluoroacetate salt formation.^[30]^ Compound **25 a** furnished the heterocycles **26 a**–**c** (61–88 %), whereas **25 b** gave **26 d**–**f** (71–98 %) and **25 c** provided **26 g** (98 % yield).

A similar sequence was employed for the preparation of the 3,5‐disubstituted compounds **30 a**,**b**. Grignard addition to the pyrrolidone intermediate **24 a** furnished the ketones **27 a**,**b** in 85–88 % yield, which were cyclized under substrate control,^[31]^ with concomitant deoxygenation to **28 a**,**b** (62–96 % yield) upon exposure to the B(ArF_5_)_3_/Ph_3_SiH reagent system.^[32]^

Further cyclization under TFA in CH_2_Cl_2_ gave the indolizidinones **29 a** ^[33]^ and **29 b** (74–99 % yield) which were employed for a new cycle of Grignard addition, hemiaminal deoxygenation, and salt formation, to furnish the trifluoroacetates **30 a**,**b** in 87–95 % yield.

Other natural products, including 5,6,8‐trisubstituted and 6,7‐dihydro IBAs were also prepared employing the same general approach.

#### 3.1.3 Withasomnine

Withasomnine (**31**) is a pyrazole derivative found in several plant species with alleged applications to a variety of ailments; the natural product is also a popular compound in alternative medicine. It is a mild analgesic and a central nervous system depressant.^[34]^ Hence, this pyrazole‐based heterocycle has elicited notorious synthetic interest.^[35]^

**Scheme 4:**
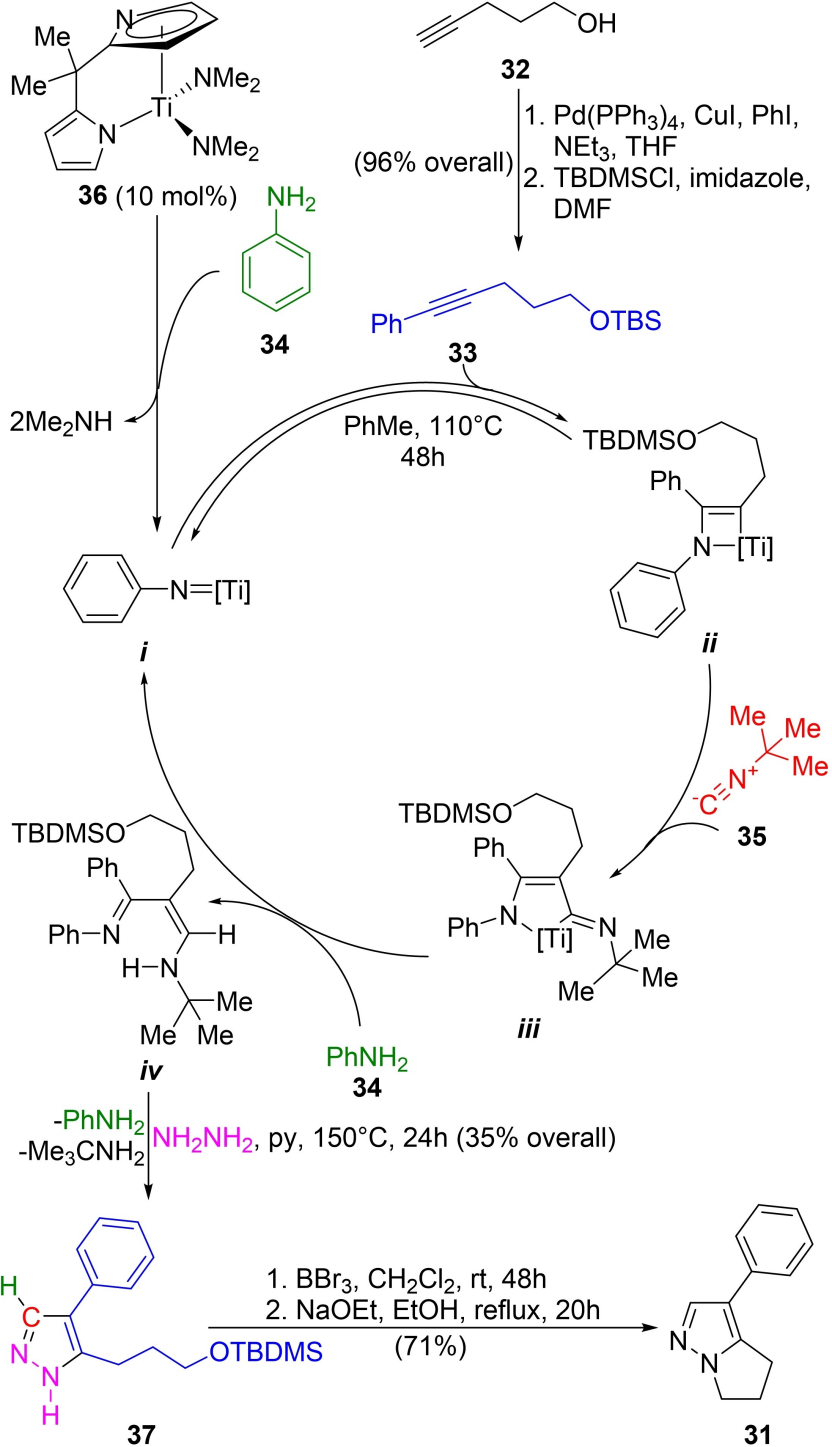
Total synthesis of withasomnine (**31**).

Odom *et al*. developed a one‐pot four‐component approach toward pyrazols, based on a Ti(IV)‐catalyzed 3CR coupling of an alkyne, an isonitrile, and a primary amine to generate unsymmetrical 1,3‐diimine tautomers, followed by removal of the volatiles and treating the crude with hydrazine in order to obtain the product.^[36]^ The approach was applied to a new total synthesis of withasomnine (Scheme 4).^[37]^

The synthesis commenced with the Sonogashira coupling of iodobenzene and 4‐pentyn‐1‐ol (**32**),^[38]^ followed by protection of the alcohol as TBDMS‐ether to afford alkyne **33** in 96 % overall yield. Next, the iminoamination of **33** in a 3CR with aniline (**34**), and *tert*‐butyl isonitrile (**35**) using 10 mol% of the titanium catalyst Ti(dpm)(NMe_2_)_2_ (**36**), followed by the addition of hydrazine hydrate gave the pyrazole **37** with the desired regioselectivity.

According to the proposed mechanism, the titanium(IV) complex, which is added as the dimethylamido‐species, acts as a precatalyst. The ligands are protolytically removed by aniline (**34**) as the primary amine substrate to generate the titanium imido complex ***i*** that initiates the multicomponent coupling. In refluxing toluene, intermediate ***i*** undergoes a [2+2] cycloaddition with the alkyne **33** and the resulting azatitanacyclobutene ***ii*** experiences the 1,1‐insertion of the isonitrile **35** to generate the 5‐membered metallacyclic intermediate ***iii***.^[39]^

In turn, in the presence of the primary amine **34**, the 5‐membered metallacycle is protolytically converted back to the titanium imido complex ***i***, releasing the iminoamination product ***iv***. Exposure of ***iv*** to hydrazine hydrate in the presence of pyridine as base afforded the pyrazole **37** in 35 % overall yield for this one‐pot procedure.

In this sequence, aniline (**34**) is fully incorporated in the reaction toward intermediate ***iv***; however, the subsequent cyclocondensation with hydrazine takes place with loss of **34** and *t*‐BuNH_2_, and only the H atom of **34** formally remains in the final product. Consequently, this case could be considered a four‐component reaction.

Removal of the TBDMS protection and subsequent conversion of the alcohol to the corresponding alkyl bromide was performed with BBr_3_, whereas the addition of NaOEt in EtOH caused the ring closure and completed the synthesis of **31** in 71 % yield.

The advantages of this catalytic multicomponent coupling strategy employed include its simplicity and the use of inexpensive and readily available reagents. In addition, the reaction can be optimized or fine‐tuned at different points, including the substituents on the isonitrile which may improve selectivity or yields and the architecture of the catalyst.

#### 3.1.4 Rigidins A–D

The rigidins A–E are marine alkaloids isolated in minute quantities (0.4–180 ppm) from the tunicate *Eudistoma cf. rigida* found near Okinawa and New Guinea.^[40]^ They were shown to display cytotoxic activity against murine leukemia L1210 cells, most likely caused by their pronounced effects on microtubule organization in cancer cells, but were inactive against HeLa (human cervical adenocarcinoma) and MCF‐7 (breast adenocarcinoma) cells. Rigidin A has also anticalmodulin activity.

**Scheme 5:**
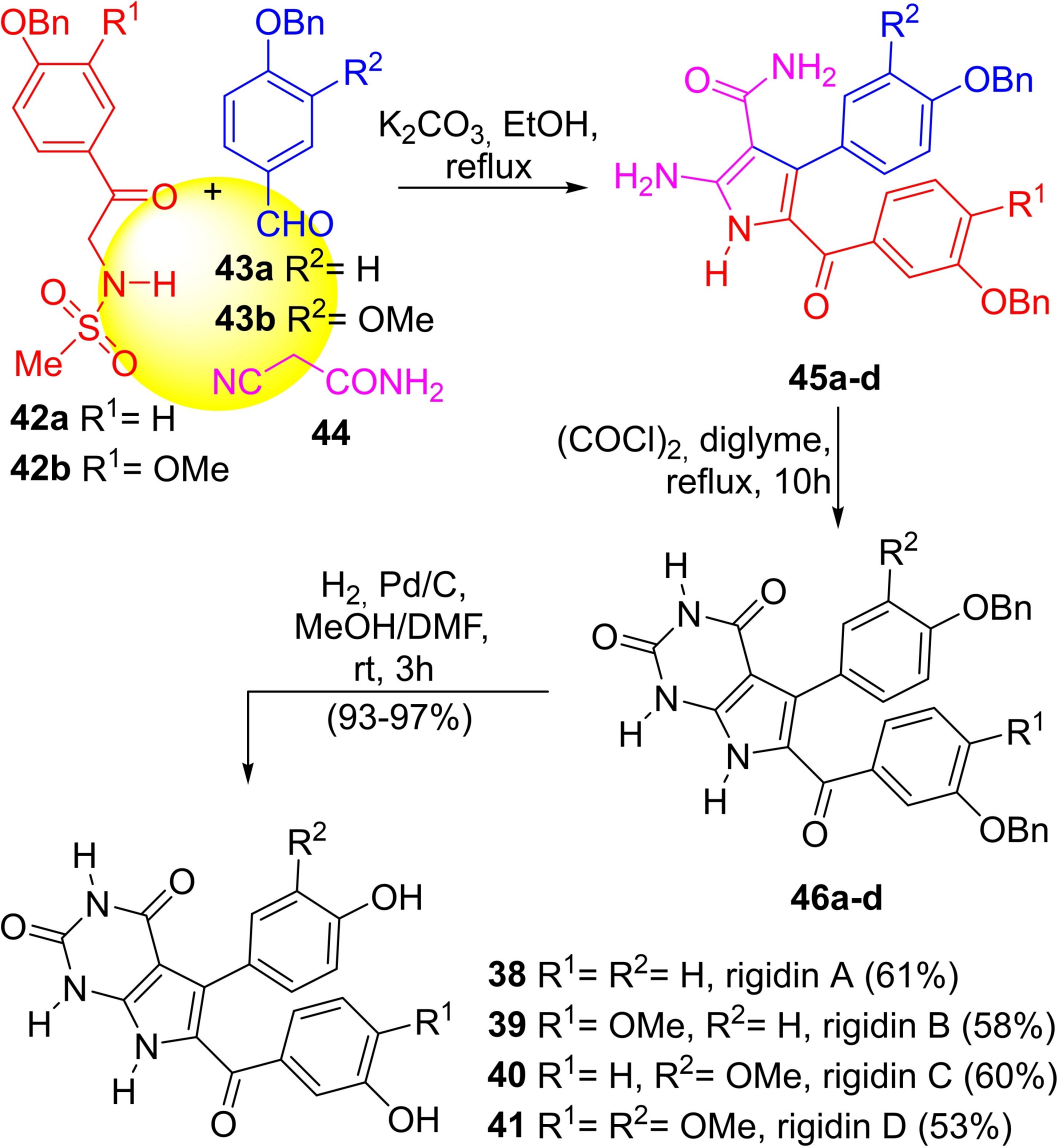
Total syntheses of the rigidins A–D (**38**–**41**).

In 2013, Kornienko and coworkers^[41]^ reported a 3CR approach toward the total synthesis of the rigidins A–D (Scheme 5).

The required sulfonamides (**42 a**,**b**) were prepared by sulfamidation of the corresponding aminoacetophenones with MsCl, and made to react with aromatic aldehydes (**43 a**,**b**) and cyanoacetamide (**44**) in the presence of K_2_CO_3_ in refluxing EtOH to give the intermediate aminopyrroles **45 a**–**d**.

The 7‐deazaxanthine skeletons were completed by carbonylation with oxalyl chloride in diglyme to give compounds **46 a**–**d**, which were further hydrogenolyzed to yield the natural rigidins (**38**–**41**) with overall yields of 53–61 % calculated from the starting aminoacetophenones. This approach represented a notorious simplification over previous syntheses.^[42]^

**Scheme 6:**
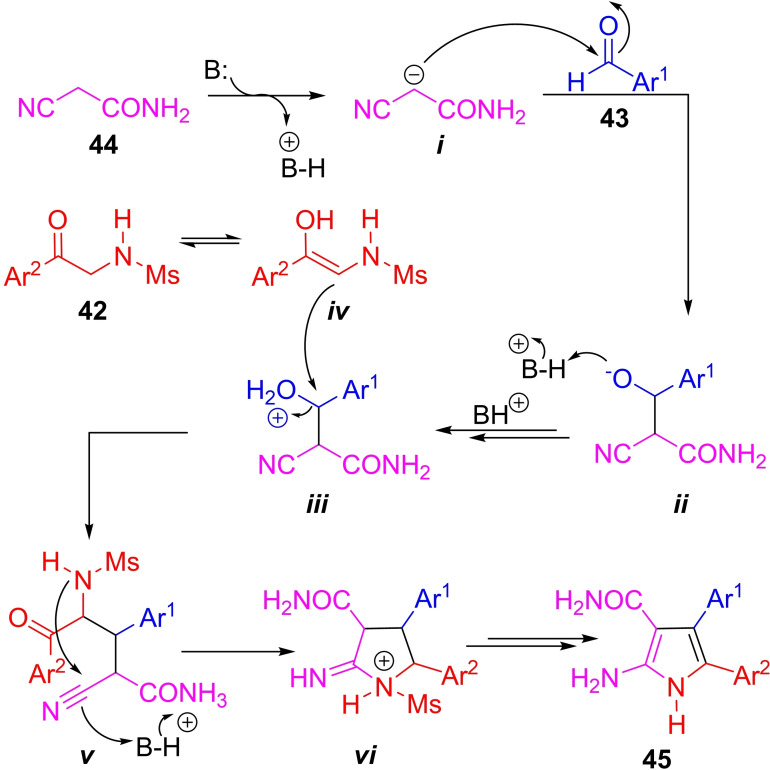
Proposed mechanism of the 3CR toward **56**, the pyrrole core of the rigidins A–D.

The formation of aminopyrrole **45** can be rationalized (Scheme 6) as taking place by the nucleophilic attack of ***i*** (formed by base‐proton abstraction of **44**) to form intermediate ***ii***. In turn, the latter would undergo protonation of the oxygen, and further activation giving rise to ***iii***, which would suffer a new nucleophilic attack by intermediate ***iv*** (formed by enolization of the starting material **42**), giving rise to ***v***. Finally, the amine moiety present in ***v*** can attack the nitrile carbon, which would give rise to the five‐membered ring ***vi***. The final steps, including elimination and demesylation, would provide the pyrrole core of the rigidins (**45**).

#### 3.1.5 (±)‐Gelliusine E

The 2,3’‐bis(indolyl)ethylamines are a small family of marine indole alkaloids characterized by their unique structures and relevant bioactivity.^[43]^ (±)‐Gelliusine E (**47**) is a racemic brominated 2,3’‐bis(indolyl)ethylamine that was isolated in 1995 by Riccio *et al*. from deep water New Caledonian sponges *Gellius* or *Orina sp*. The natural product was isolated along with the related gelliusine F,^[44]^ and two racemic mixtures of diastereomeric brominated trisindoles [(±)‐gelliusine A and B], (±)‐gelliusine C, (±)‐gelliusine D.

These alkaloids possess anti‐serotonin activity, and (±)‐gelliusine E displayed a strong affinity for somatostatin and neuropeptide Y receptors, and proved to be a potent inhibitor of specific ligand binding in the human B2 bradykinin receptor‐binding assay.

**Scheme 7:**
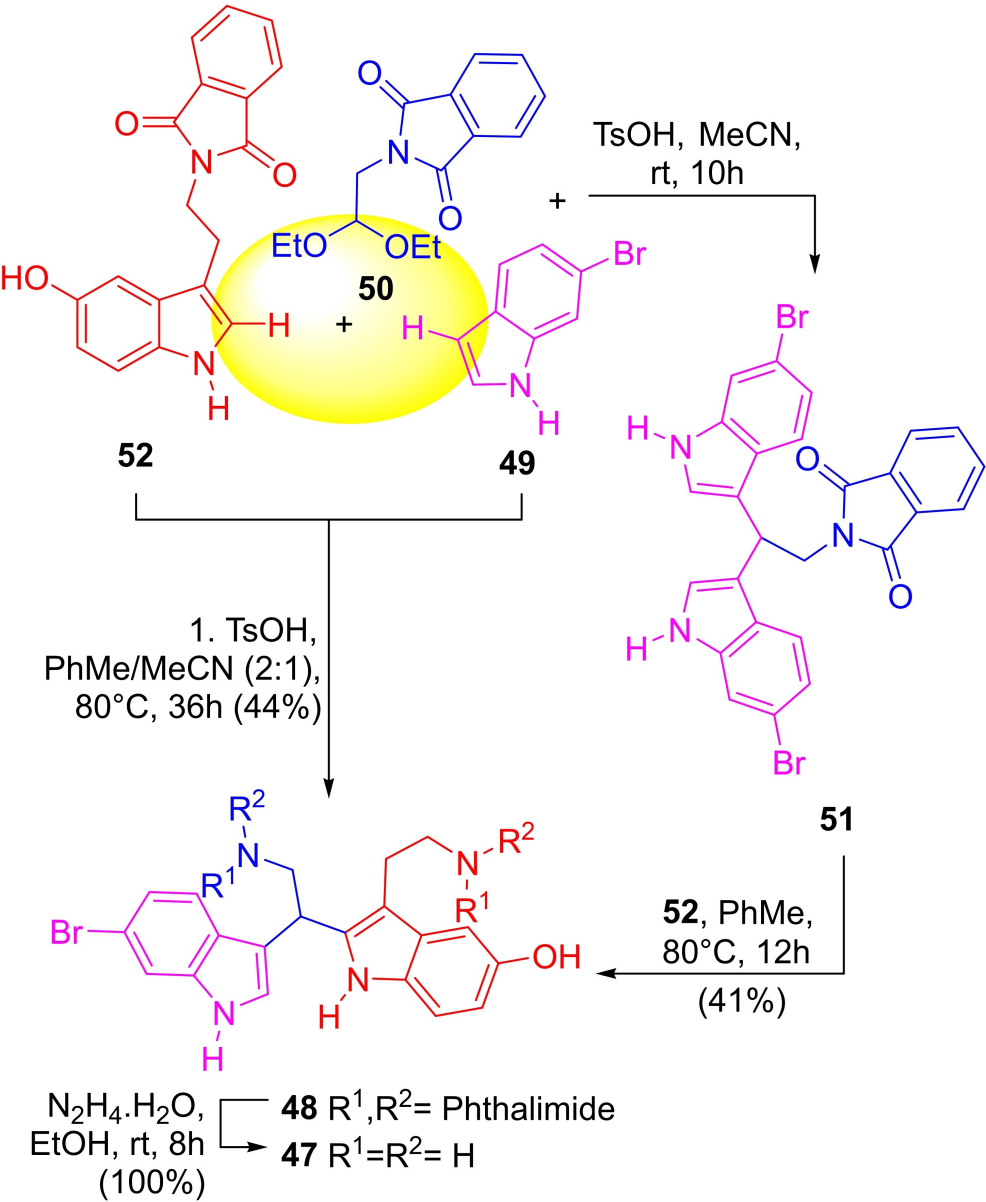
Total synthesis of gelliusine E (**47**).

In 2012, the group of Jaratjaroonphong reported a one‐pot sequential two‐step total synthesis of *N*‐protected gelliusine E (**48**),^[45]^ based on the TsOH‐catalyzed transindolylation of a bis‐indolylmethane derivative. The first step (Scheme 7) involved the diindolylation of 6‐bromoindole (**49**) with the protected diethylamino acetal **50** ^[46]^ under TsOH catalysis^[47]^ in MeCN at room temperature for 10 h. The so‐produced intermediate precipitated as a yellow solid, which was treated *in situ* with a solution of the protected tryptamine **52** in toluene. Refluxing the reaction at 80 °C for 12 h gave the bis‐phthalimide of the natural product in 50 % yield.

They also disclosed a one‐pot three‐component strategy toward the natural product with TsOH in PhMe/MeCN, which afforded the protected natural product (**48**) in 44 % yield. In both cases, hydrazinolysis of the latter afforded **47** quantitatively. A mechanism to account for the reaction was also proposed, based on literature precedents.^[48]^

#### 3.1.6 Manzamine A and Related Compounds: Ircinol, Ircinal, and Methyl Ircinate

Manzamine A (**53**) was first reported in 1986 by Higa *et al*., as a result of a bio‐guided search aimed to find cytotoxic substances in the marine sponge *Haliclona sp*. harvested in Cape Manzamo (Okinawa, Japan),^[49]^ where tiny amounts of **53** were obtained (1.4×10^−2^ %*w/w*).

The natural product showed inhibition of P388 mouse leukemia cells at low micromolar level (IC_50_=0.07 μg/mL), being currently investigated as a lead molecule for the treatment of neoplasms, with a promising horizon.^[50]^ It has also been shown to display antibacterial, anti‐HIV, and anti‐inflammatory properties.^[51]^

**Scheme 8:**
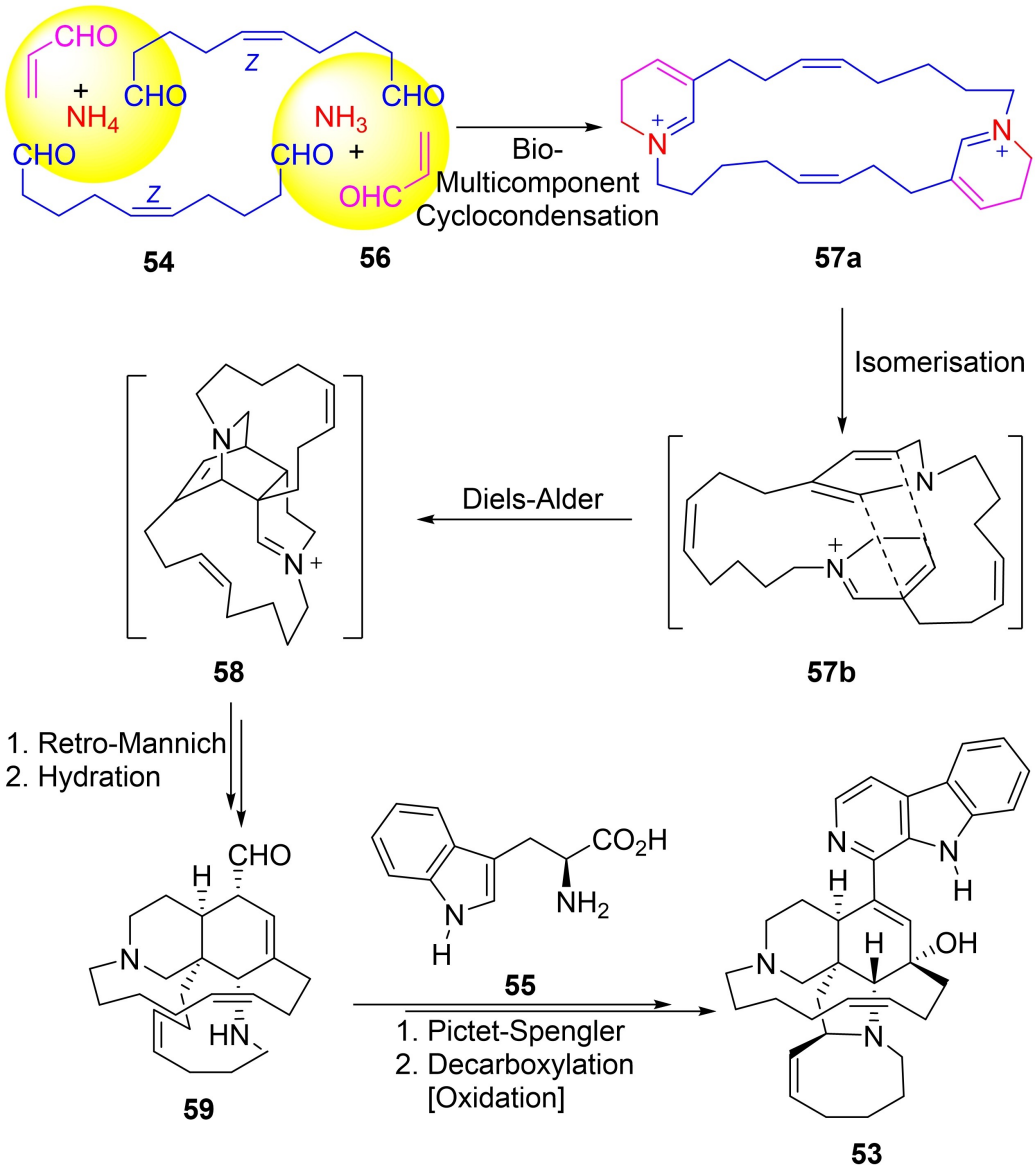
Bio‐MCR‐based proposed biosynthesis of manzamine A (**53**).

The biosynthesis of manzamine A is partially known and its critical key steps were communicated in 1992 by Baldwin *et al*.^[52]^ In their study, it was proposed (Scheme 8) that a biological precursor could be dissected into four building blocks, including NH_3_, a symmetrical dialdehyde C10 unit (**54**), tryptophan (**55**), and an acrolein bioequivalent (**56**, C3 unit) by an *in vivo* multicomponent reaction to afford the adduct **57 a**, which after isomerization would provide the intermediate **57 b**.

The key step in the proposal was an intramolecular *endo* Diels‐Alder cycloaddition of the bis‐dihydropyridine **57 b** to provide the tetracycle **58**, under an unknown Diels‐Alderase.^[53]^ Further reactions on **58** would afford the formyl intermediate **59** which after the final incorporation of tryptophane (**55**), followed by hydration and further oxidative steps would afford the full molecular architecture of manzamine.^[54]^

**Scheme 9:**
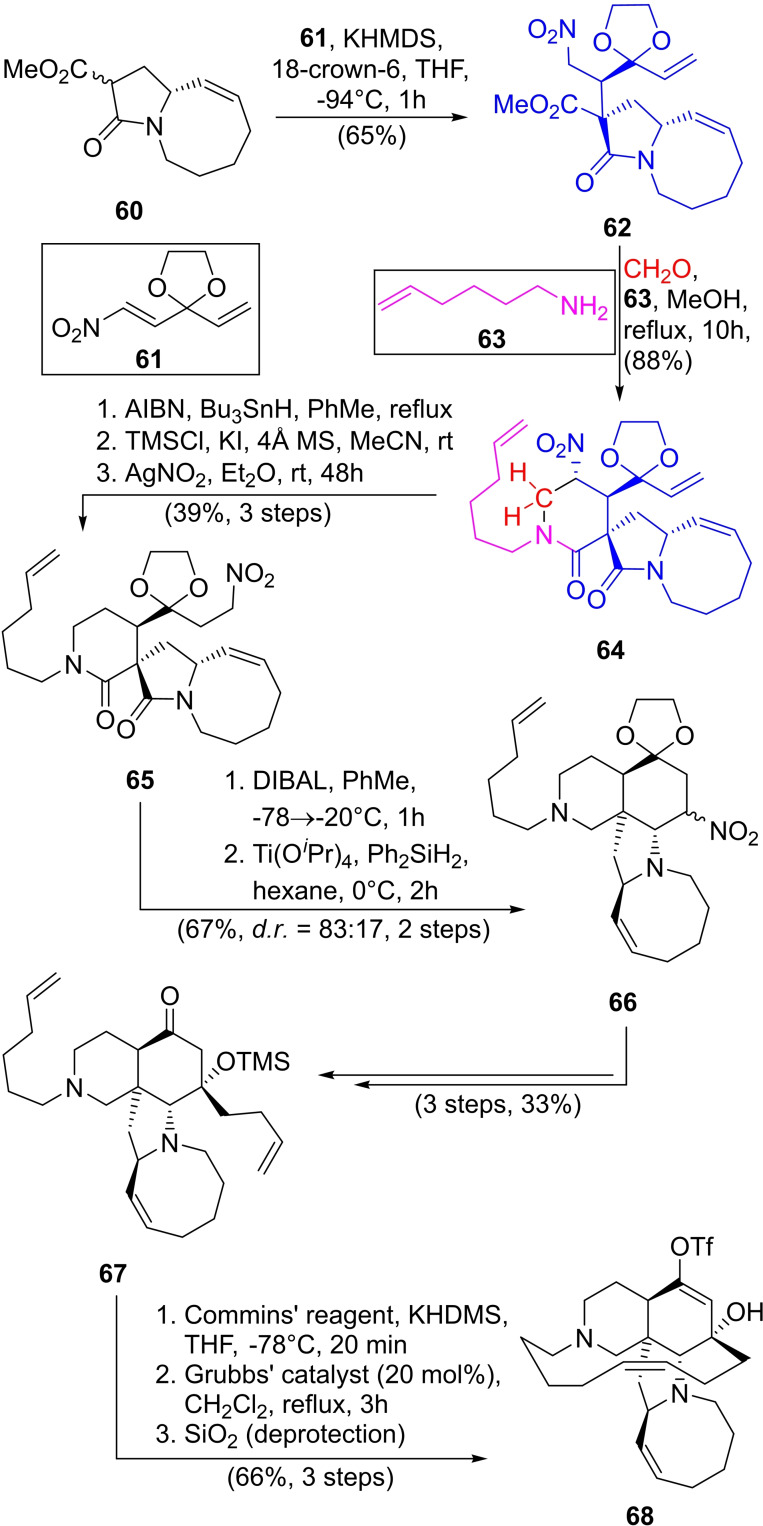
Synthesis of the key intermediate **68** toward the family of manzamine alkaloids.

After 12 years since its discovery, the first total synthesis of manzamine A was reported by Winkler *et al*. encompassing a complex and challenging process that involved *ca*. 40 steps to render this marine natural product.^[55]^ Since this pioneering work, several other (formal) total syntheses were reported; Interestingly, however, the group of Dixon developed an approach entailing a 3CR to synthesize the piperidin‐2‐one ring of the key intermediate **64** (Scheme 9).^[56]^

In this work, the precursor **62** was obtained by a Michael addition of the β‐ketoester **60** and the Michael acceptor **61** using KHMDS with 18‐crown‐6 (65 % yield, *dr*=3 : 1), and used to perform a 3CR reaction, which involves a nitro‐Mannich/lactamization^[57]^ cascade with formaldehyde and the amine **63**. This transformation resulted in 4‐nitro‐piperidin‐2‐one **64** in 88 % yield.

Subsequent steps consisted of nitro group removal, the transformation of the alkene into the terminal iodide followed by nitro functionalization to give the intermediate **65** in 39 % yield in three steps. In turn, **65** was carefully reduced to the lactam with DIBAL, followed by a further intramolecular Mannich reaction under Buchwald conditions,^[58]^ affording tetracycle **66** in 67 % overall yield and high diastereofacial selectivity (83 : 17).

The next steps focused on the transformation of the nitro moiety to a carbonyl group with TiCl_3_ and employing a modified Neff reaction.^[59]^ The addition of 3‐butenylmagnesium bromide to the carbonyl, followed by silyl protection of the resulting alcohol and acetal deprotection furnished intermediate **67** in 33 % overall yield (four steps).

**Scheme 10:**
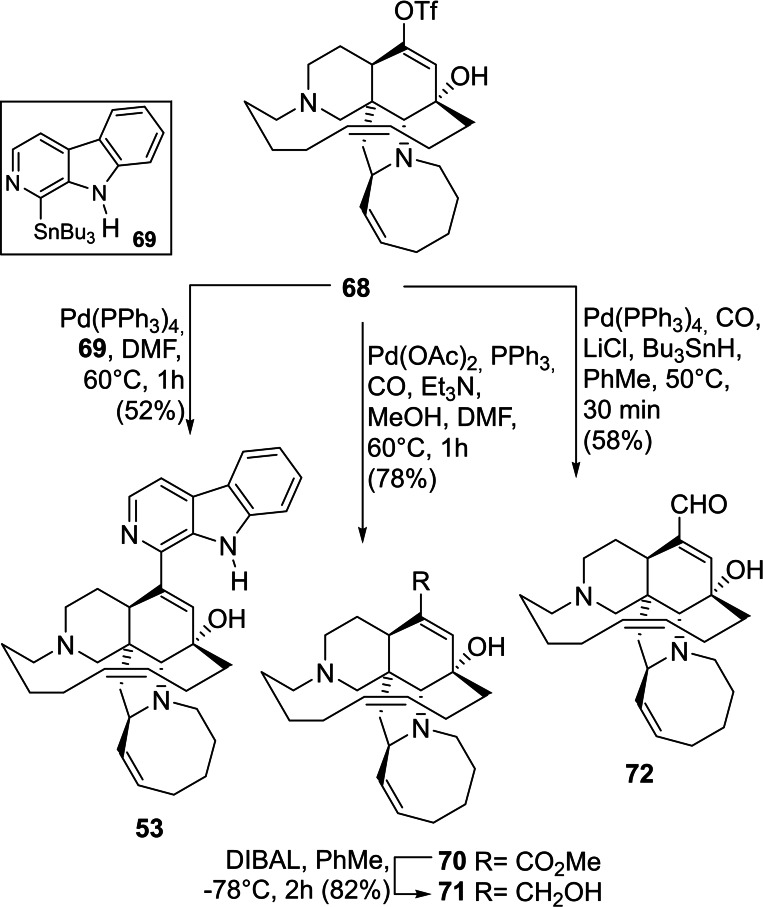
Total syntheses of manzamine A (**53**), methyl ircinate (**70**), ircinol A (**71**), and ircinal A (**72**). The final steps.

Formation of the enol triflate using Commin's reagent followed by ring closing metathesis under Grubbs I catalysis, gave the central intermediate **68** in 66 % overall yield in two steps. In turn, **68** was subjected to different palladium‐catalyzed cross‐coupling functionalizations to obtain different natural products of this family (Scheme 10).

The cross‐coupling with stannyl β‐carboline **69** produced manzamine A (**53**) in 52 % yield, while carbonylation gave 78 % yield of methyl ircinate (**70**), whose ester reduction provided ircinol A (**71**) in 82 % yield. Finally, carbonylation under reductive conditions with Bu_3_SnH produced ircinal A (**72**) in 58 % yield.

Interestingly, a nitro‐Mannich reaction was also employed as a key step for the total synthesis of nakadomarin A, a cytotoxic manzamine‐type alkaloid isolated from the sponge *Amphimedon sp*.^[60]^

#### 3.1.7 (±)‐Exotine B

Plants of the genera Murraya are part of Traditional Chinese Medicine, where their roots and leaves are prescribed to treat inflammatory diseases, such as rheumatism, eczema, and abdominal pain.^[61]^ Exotine B (**73**) is a cyclohepta[*b*]indole natural product, isolated from the roots of *Murraya exotica*,^[62]^ that can be regarded as a very rare heterodimer of isopentenyl‐substituted indole and coumarin derivatives.^[63]^ Biosynthetically, it seems to be derived from gleinadiene (**74**)^[64]^ and a tautomer of a 3‐indole diene, a dimer of which has been isolated from the same plant.^[65]^ The natural product inhibits the production of nitric oxide in lipopolysaccharide‐induced BV‐2 microglial cells.

**Scheme 11:**
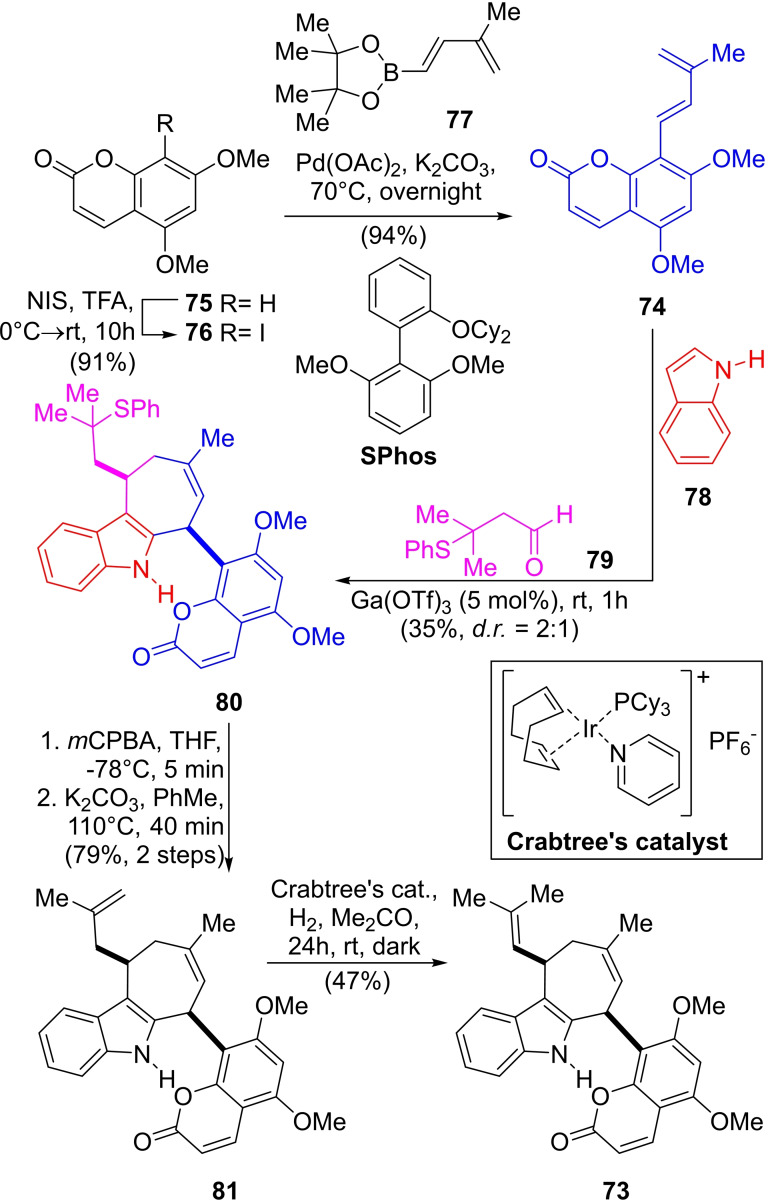
Total synthesis of (±)‐exotine B (**73**).

In 2018, the group of Trauner reported the first total synthesis of **73**,^[66]^ under the conditions developed by Wu *et al*.^[67]^ based on a 3CR catalyzed by Ga(OTf)_3_ between indole (**78**), the diene **77**, and the aldehyde **79** as a surrogate for prenal (Scheme 11), due to the comparatively poor reactivity of this α,β‐unsaturated aldehyde.^[68]^

The key component gleinadiene (**74**) was obtained in 86 % total yield,^[69]^ under mercury‐free conditions,^[70]^ and in a multigram scale, by regioselective iodination of 5,7‐dimethoxy coumarin (**75**) with *N*‐iodosuccinimide under TFA activation, followed by a Suzuki cross‐coupling of the iodide **76** with the boronate ester **77** under Buchwald's conditions^[71]^ to furnish **74** in 94 % yield on a multigram scale.

After optimization, the key Ga(OTf)_3_ catalyzed [4+3] cycloaddition reaction furnished the projected intermediate **80** in a modest 35 % yield (*dr*=2 : 1) but was amenable for scale‐up and afforded the major isomer **80** by crystallization.

Installation of the trisubstituted double bond entailed a three‐step process where the thioether of **80** was selectively oxidized with *m*CPBA and the resulting sulfoxide was thermally eliminated in refluxing toluene in the presence of K_2_CO_3_ as the base to afford the terminal olefin **81** (iso‐exotine B) as a 7 : 1 mixture with exotine B (**73**). Finally, a carefully controlled reaction with Crabtree's catalyst in acetone caused the required olefin isomerization to furnish **73** in 47 % yield.

### 3.2 Oxygen‐Containing Natural Products

This section discusses the 3CR‐based total syntheses of natural products bearing oxygen heterocycles, in the form of cyclic ethers, lactones or both features.

#### 3.2.1 Isourolithin A

The dibenzo‐α‐pyrone framework is a common structural motif found in many biologically relevant natural products.^72^ Isourolithin A (**82**) is a dibenzo‐α‐pyrone type intestinal microbial metabolite of ellagitannins and ellagic acid. It is also a potential inhibitor of 3‐phosphoglycerate kinase, which also displays antiproliferative activity.^[73]^ It can contribute significantly to the beneficial properties attributed to its precursors.

The composition of the gut microbiota impacts its ability to produce isourolithin A; hence, the health benefits associated with the consumption of ellagitannins differ considerably among individuals. Very recently, different groups informed the isolation of human bacteria capable of producing isourolithin A.^[74]^

The group of Tamperini designed and systematically optimized a multicomponent acid‐catalyzed enolacetylation of (hetero)arylidene acetones followed by a metal‐free Diels‐Alder reaction with an electron‐poor alkyne, such as dimethyl propiolate,^[75]^ as a useful strategy toward functionalized biaryls.

**Scheme 12:**
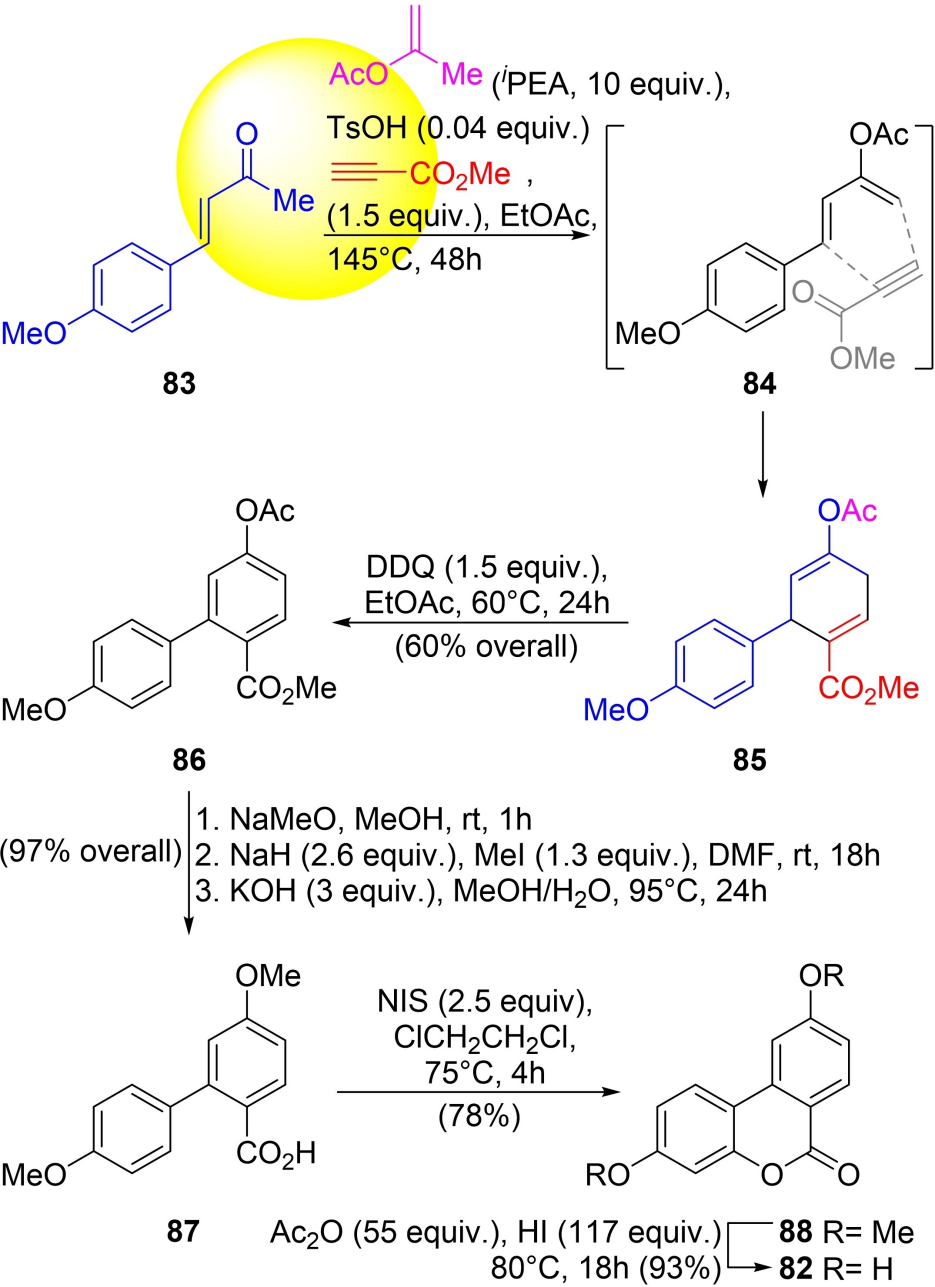
Total synthesis of isourolithin A (**82**).

The use of methyl propiolate as a dienophile in metal‐free Diels‐Alder reactions is interesting, due to the scarce reactivity of this compound which results in poor reaction yields.^[76]^ The reaction, which should be performed in a metal vessel, affords a cyclohexadiene oxidation product, which can be further oxidized to obtain the corresponding aromatic products (Scheme 12).^[77]^

The strategy was employed in 2019 by the group of Temperini for the total synthesis of isourolithin A, as an alternative to the previous synthesis of the natural product, made by the condensation of resorcinol with 4‐hydroxy‐substituted 2‐bromobenzoic acid in an alkaline medium under CuSO_4_ catalysis (Hurtley reaction).^[78]^

In this recent approach, the reaction of 4‐methoxy‐arylbutenone **83** (prepared by the aldol condensation of *p*‐anisaldehyde with acetone)^[79]^ with isopropenyl acetate (^*i*^PEA) and methyl propiolate in EtOAc at 145 °C gave **85** through **84** in a Diels‐Alder reaction with methyl propiolate. The cyclized product was oxidized with the recyclable oxidant DDQ^[80]^ at 60 °C in EtOAc to deliver the biaryl derivative **86** in 60 % overall yield for the enolization‐cycloaddition‐aromatization process.

Next, the acetoxy‐protecting group was removed and the free phenol was methoxylated with MeI, using NaH as the base. Basic hydrolysis of the carbomethoxy group completed the sequence, to afford the acid **87** in 97 % overall yield.^[81]^

The *o*‐phenyl benzoic acid **87** was oxidized by treatment with NIS in dichloroethane^[82]^ followed by cyclization to afford dimethyl isourolithin A (**88**) in 78 % yield. The final dealkylation of the methyl ethers with HI gave the natural product (**82**) in 93 % yield.^[83]^

#### 3.2.2 (+)‐Rottnestol

(+)‐Rottnestol (**89**) was isolated in 1995 by Erickson *et al*. from marine sponges of the genus *Haliclona* harvested near Rottnest island (Western Australia), during their studies of cytotoxic natural products. The natural product, which is devoid of cytotoxicity, exhibits a long polyalkenyl chain and a terminal hemiketal‐tetrahydropyran ring.^[84]^

Compound **89** was almost inactive against some bacterial strains (*B. subtilis, E. coli*, and *M. luteus*) and fungi (*G. graminis* var*. tritici, P. grisea, C. albicans*, and *S. cerevisiae*), but effectively inhibited different weeds growing in wheat at a load of 0.069 kg/Ha, showing little to no phytotoxicity against soybean, rice, and wheat.

**Scheme 13:**
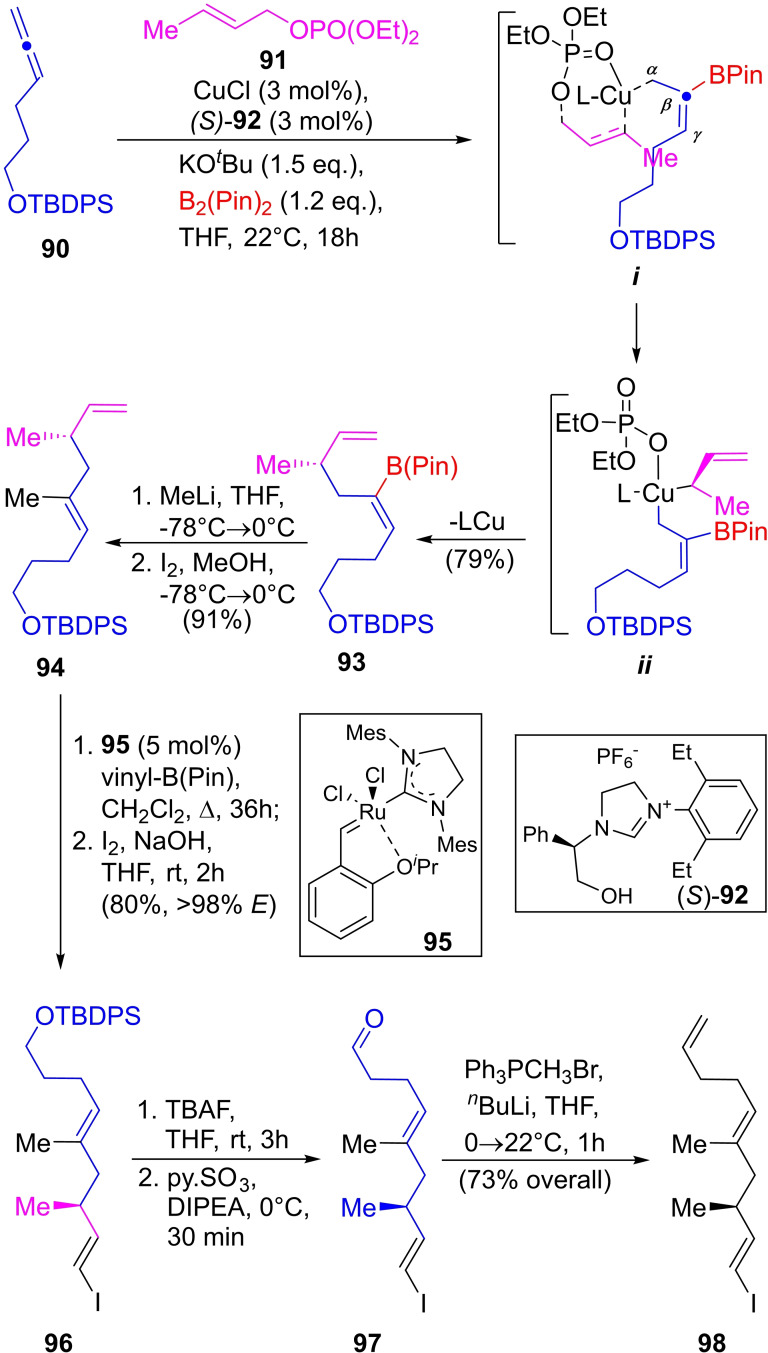
Synthesis of the vinyl iodide intermediate **98** toward rottnestol, featuring an enantioselective allylic substitution (EAS) methodology.

The first total synthesis of (+)‐rottnestol was reported in 1999,^[85]^ and in 2014 the group of Hoveyda published a multi‐gram scalable multicomponent approach toward the natural product, showcasing its applicability.^[86]^ Method optimization for the enantioselective allylic substitution (EAS) enabled access to a series of trisubstituted alkenes with high diastereoselectivity and under mild conditions (Scheme 13).

In particular, this technique was applied to the synthesis of the (*Z*)‐alkenyl boronate **93** from allene **90** protected as *tert*‐butyldiphenylsilyl ether (TBDPS) and allylic phosphate **91**, where the addition of CuCl and B_2_(Pin)_2_ served to form the Cu−B(pin) species, capable to perform an α addition of Cu and a β attack of BPin on the allene derivative.

The formation of the C−C bond takes place through a ligand‐mediated double stereocontrolled cooper coordination. The initial coordination with the oxygen of the phosphonate facilitates the coordination with the allylic carbon (***i***) and the chiral ligand (*S*)‐**92**. The intermediate ***i*** undergoes transformation into the square planar Cu(III) complex ***ii***. In turn, the C−C bond is rapidly formed through a reductive elimination, affording the product (*S*)‐**93** with exquisite stereocontrol.

The transformation allowed access to **93** in 98 % yield (*E : Z*=2 : 98; *er*=92 : 8). The reaction of **93** with MeLi in THF and iodine performed methylation with concomitant configurational inversion of the alkene providing **94** in 91 % yield (*E : Z*>98 : 2). Next, vinyl‐borylation by olefin cross‐metathesis followed by iodination gave **96** with 80 % overall yield (*E*>98 %). Subsequently, desilylation and mild oxidation to the aldehyde intermediate (**97**), followed by Wittig vinylation with Ph_3_PCH_3_Br produced the triene **98** in 73 % overall yield.

**Scheme 14:**
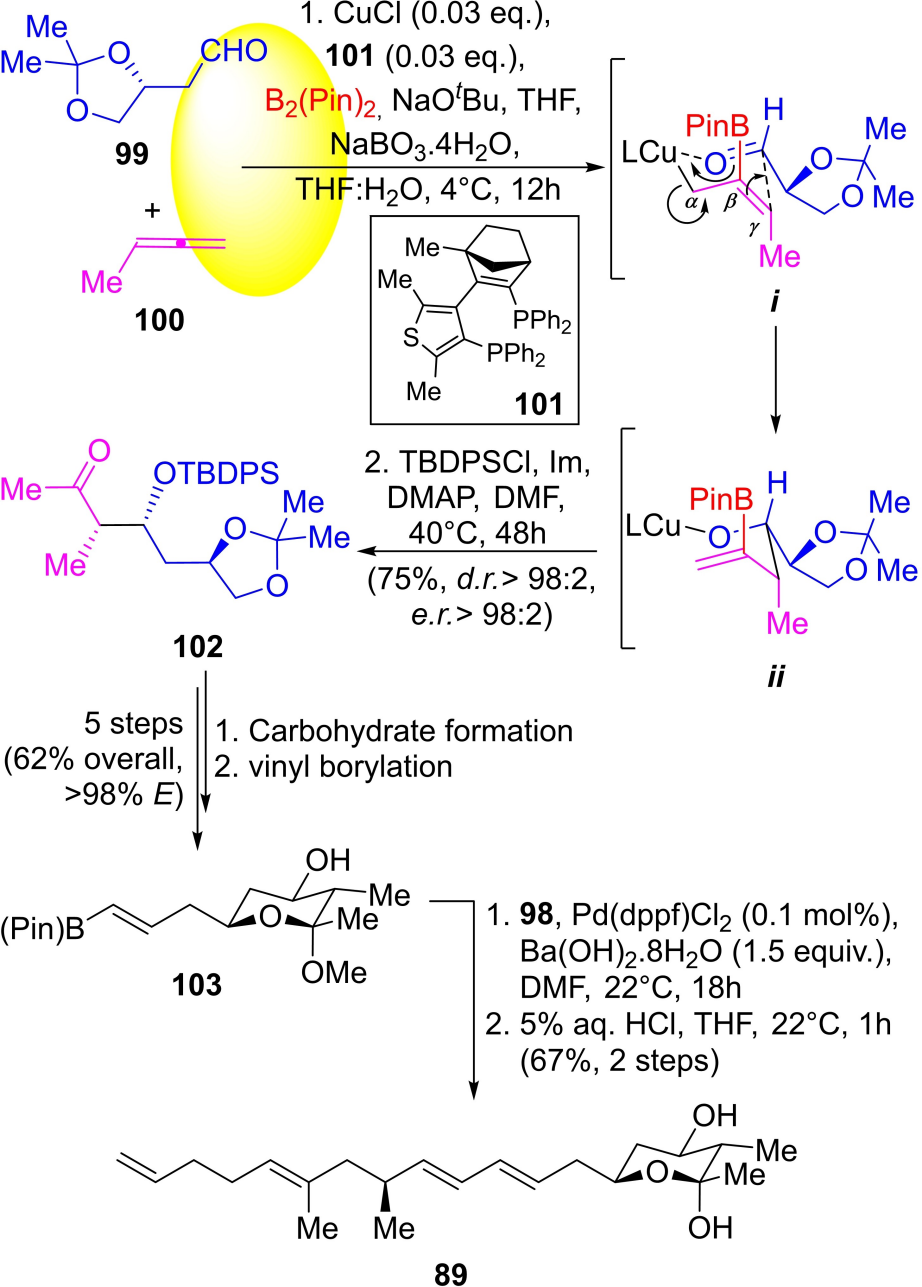
Concluding steps of the total synthesis of (+)‐rottnestol (**89**).

The preparation of the carbohydrate building block involved the treatment of the allene **100** with the aldehyde **99** under boryl‐copper conditions (Scheme 14) catalyzed by **101**. The initial formation of the organo‐copper intermediate ***i*** resulted from the α,β‐addition of boryl‐Cu species to allene **100**. In turn, intermediate ***i*** coordinated to the carbonyl oxygen of aldehyde **99**, providing the stacked species ***ii***.

The formation of new C−C (at the γ position) and O−Cu bonds, allowed the creation of a vinylboronate moiety in the intermediate ***ii***. Perborate promoted oxidation of a C(*sp* ^2^)‐BPin bond to an O−C(*sp* ^2^) bond provided the enol form of the so‐formed β‐hydroxy‐ketone. In the sequence, the secondary carbinol was masked as a *t*‐butyl‐diphenylsilylether to produce protected compound **102** (75 % yield, *er*>98 : 2).

Afterward, it was transformed into an alkyne carbohydrate, where the alkyne moiety was converted to the pinacol borate vinyl ester **103** in 62 % overall yield (*E*>98 %) in five steps. Finally, **103** was cross‐coupled with vinyl iodide **98** giving (+)‐rottnestol (**89**) in 67 % overall yield after hemiacetal formation.

#### 3.2.3 Herboxidiene

Herboxidiene (**104**, GEX1 A) was discovered in 1991 by Miller‐Wideman *et al*. from *Streptomyces chemofuscus* A7847, during a screening of fermentation broths obtained from soil samples collected at Stepping Stone Falls (Rhode Island, USA), aimed to find new herbicidal compounds, and has been the subject of different synthetic efforts.^[87]^

Herboxidiene is a potent antitumor agent that targets the SF3B subunit of the spliceosome.^[88]^ Increasing evidence points to mutations in SF3B1 of the spliceosome and their involvement in various types of human cancers, including hematological malignancies and solid tumors.^[89]^

**Scheme 15:**
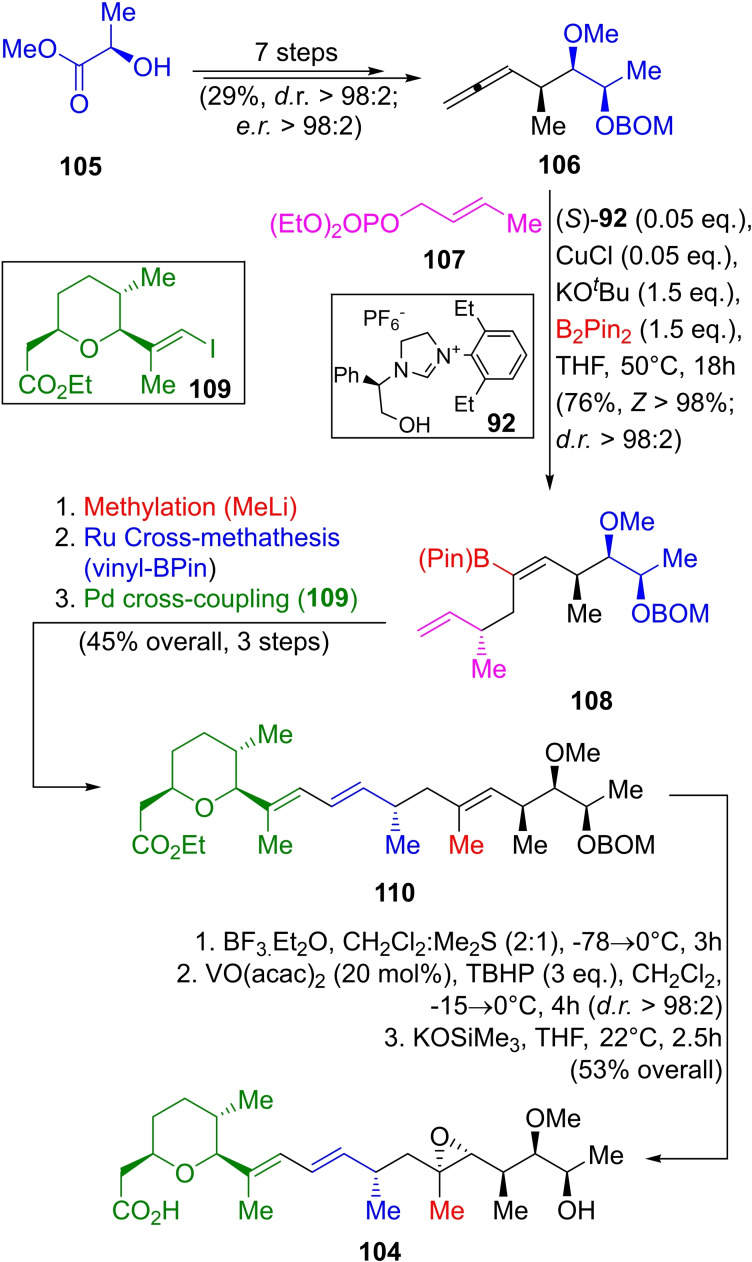
Three‐component EAS approach toward herboxidiene (**104**).

The total synthesis of herboxydiene (Scheme 15) required to construct a more complex allene **106**, which was synthesized from (*R*)‐methyl lactate (**105**).^[86]^ Applying the same three‐component catalytic EAS methodology with **106** and the allylic phosphate **107**, the pinacolyl‐vinylboronate **108** was obtained with a 76 % yield and exquisite stereochemistry (*Z*>98 %, *dr*=98 : 2).

This boronic ester was methylated, and a cross‐metathesis produced the corresponding vinyl boronate, which was treated with the vinyl iodide derivative **109** [prepared in 8 steps from β‐(+)‐citronellene] giving a 43 % overall yield of the intermediate **110** in three steps.

Finally, **110** was subjected to BOM deprotection under BF_3_.Et_2_O promotion, epoxidation with the VO(acac)_2_/TBHP reagent system, and saponification with potassium trimethylsilanoate to produce the natural product **104** in 53 % overall yield for the last three steps.

#### 3.2.4 Psiguadial B

Psiguadial B (**111**) is a complex meroterpenoid that contains a bicyclo[4.3.1]decane ring system *trans*‐fused to both, a cyclobutane and a highly functionalized chromane. It was first isolated in minute quantities (25 mg from 20 Kg of leaves) by Ye and co‐workers in 2010^[90]^ from *Psidium guajava*, a fruit‐bearing tree that is used in traditional medicine systems of Central America, the Caribbean, Africa, and Asia for the treatment of different conditions, including diarrhea and hyperglycemia.^[91]^

The natural product has antitumor activity, with high selectivity for HepG2 over HepG2/ADM cells [IC_50_=45.6 : 1.4 nM and 25.1:0.2 μM, respectively], probably acting as an inhibitor of P‐glycoprotein.

**Scheme 16:**
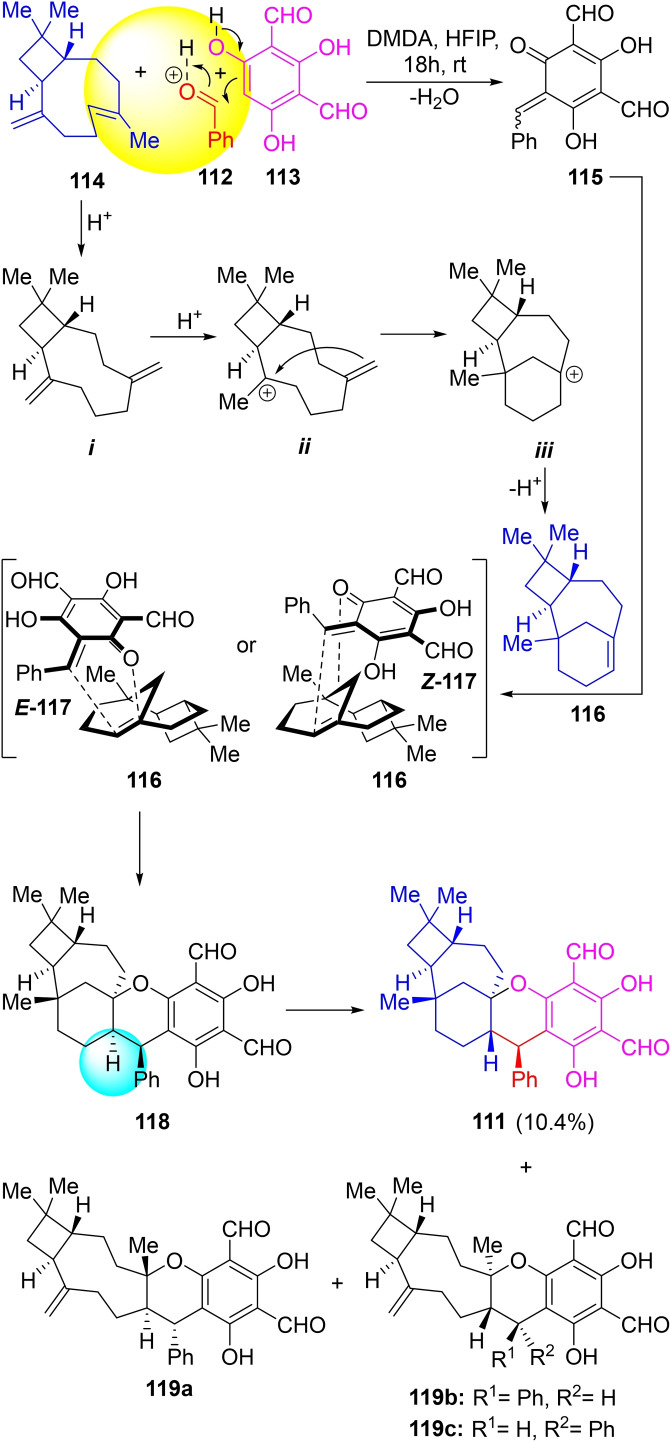
Total syntheses of psiguadial B (**111**) and a hetero‐Diels‐Alder based mechanistic proposal for its 3CR formation from **112**, **113** and **114**.

In 2017, the group of Cramer reported a one‐step multigram‐scale biomimetic approach to psiguadial B^[92]^ based on the 3CR between benzaldehyde (**112**), diformyl phloroglucinol (**113**), and caryophyllene (**114**), which involves the construction of two rings and four stereocenters (Scheme 16).

After an optimization process, it was found that HFIP (hexafluoroisopropyl alcohol), being polar and less nucleophilic than trifluoroethyl alcohol (TFE) was the best solvent to promote the formation of cationic intermediates, whereas *N,N’*‐dimethylethylenediamine (DMEDA) proved superior as the reaction catalyst.

The transformation was performed open to the air and at ambient temperature, affording **111** in 10.4 % yield, in a mixture with other compounds [**119 a**, guajadial (**119 b**) and psidial (**119 c**)], totaling 37 %, which could not be converted into **111** even in the presence of excess benzaldehyde and caryophyllene.

Different mechanisms, based on hetero‐Diels‐Alder (hDA), Michael addition, and Alder‐ene reactions were proposed to explain the biomimetic formation of **111**. The hDA reaction alternative is illustrative. It involves the reaction of the known terpene **116** with the geometric isomers of the reactive *ortho*‐quinonemethide (*o*‐QM) **117** (Scheme 16), formed by *in situ* condensation of benzaldehyde (**112**) with the phenol **113**, to afford 9‐*epi*‐psiguadial B (**118**), which features a *cis*‐fused chromane. The final acid‐catalyzed isomerization of this ring junction would provide the natural product (**111**).

It was also proposed that **118** is generated through an initial acid‐catalyzed isomerization of the more reactive (endocyclic) double bond of caryophyllene (**114**) to afford the exocyclic isomer ***i***. In turn, this could undergo protonation toward ***ii*** and a diastereoselective intramolecular cationic alkene cyclization to furnish the carbocation ***iii***. Final selective deprotonation of the tricycle ***iii*** would provide **116**.

In addition, computational studies helped to hypothesize that the biosynthesis of **111** may proceed through the Michael addition of caryophyllene on the *ortho*‐quinonemethide intermediate **117** ^[93]^ derived from diformyl phloroglucinol, followed by a series of proton transfers and cationic cyclizations, without involving any enzyme. Noteworthy, the simplicity and efficiency of the 3CR contrasted with Resiman's total synthesis of the natural product, reported in 2016, which proceeded in 16 steps through an abiotic approach and gave the natural product on a less‐than 10 mg scale.^[94]^

#### 3.2.5 (+)‐Linoxepin

Linoxepin (**120**) is a unique lignan isolated in 2007 by Schmidt *et al*. from the aerial parts of *Linum perenne* L. (Linaceae).^[95]^ This chiral lactone is a six‐ring caffeic acid dimer, which features a dibenzo‐dihydrooxepine moiety fused to an oxidation‐prone dihydronaphthalene core.

**Scheme 17:**
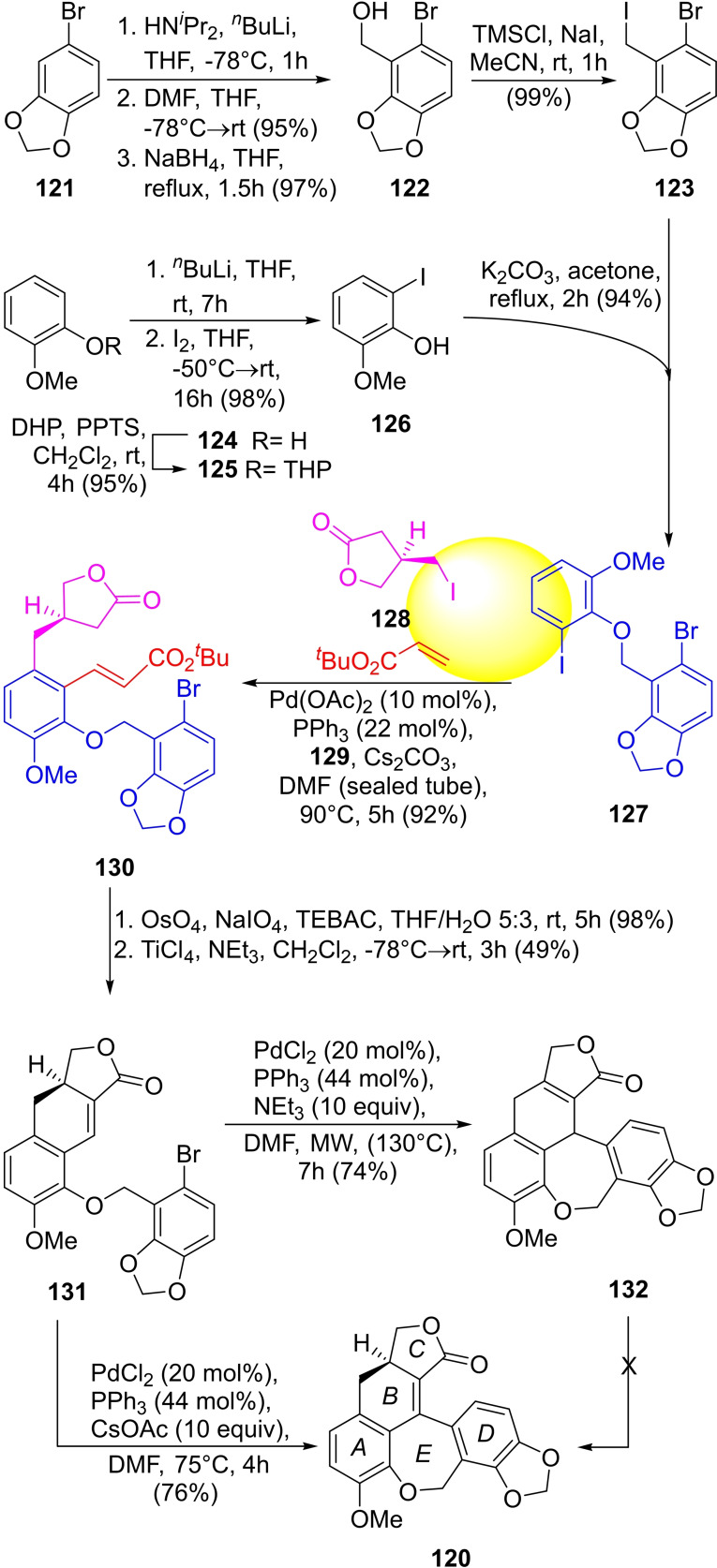
Catellani‐mediated total synthesis of (+)‐linoxepin (**120**).

The group of Lautens reported in 2013 total syntheses of (+)‐linoxepin and its racemic counterpart,^[96]^ using a transition‐metal‐catalyzed Catellani domino reaction as the key strategy (Scheme 17). The critical fragment **127** required for the Catellani reaction was prepared in 94 % yield by a Williamson etherification of the phenol **126** with the benzyl iodide **123**. The former was synthesized in 93 % overall yield from guaiacol (**124**), by tetrahydropyranylation of its phenol moiety, followed by *ortho*‐metallation of the resulting THP‐ether **125**, iodination with I_2,_ and deprotection.

On the other hand, **123** was easily accessed from **121** by means of a heteroatom‐directed *ortho*‐metallation with LDA in THF, followed by formylation with a DMF quench of the metallated species, reduction of the formyl moiety to alcohol **122** (95 % yield) and final transformation of the latter to the corresponding iodide **123** in 98 % yield, with the NaI/TMSCl reagent.

The iodoarene **127** was submitted to a Catellani reaction with *tert*‐butyl acrylate and iodolactone **128**, prepared in both, racemic and chiral forms, according to Zutter *et al*.,^[97]^ to afford the lactonic key intermediate **130** in 92 % yield.

**Scheme 18:**
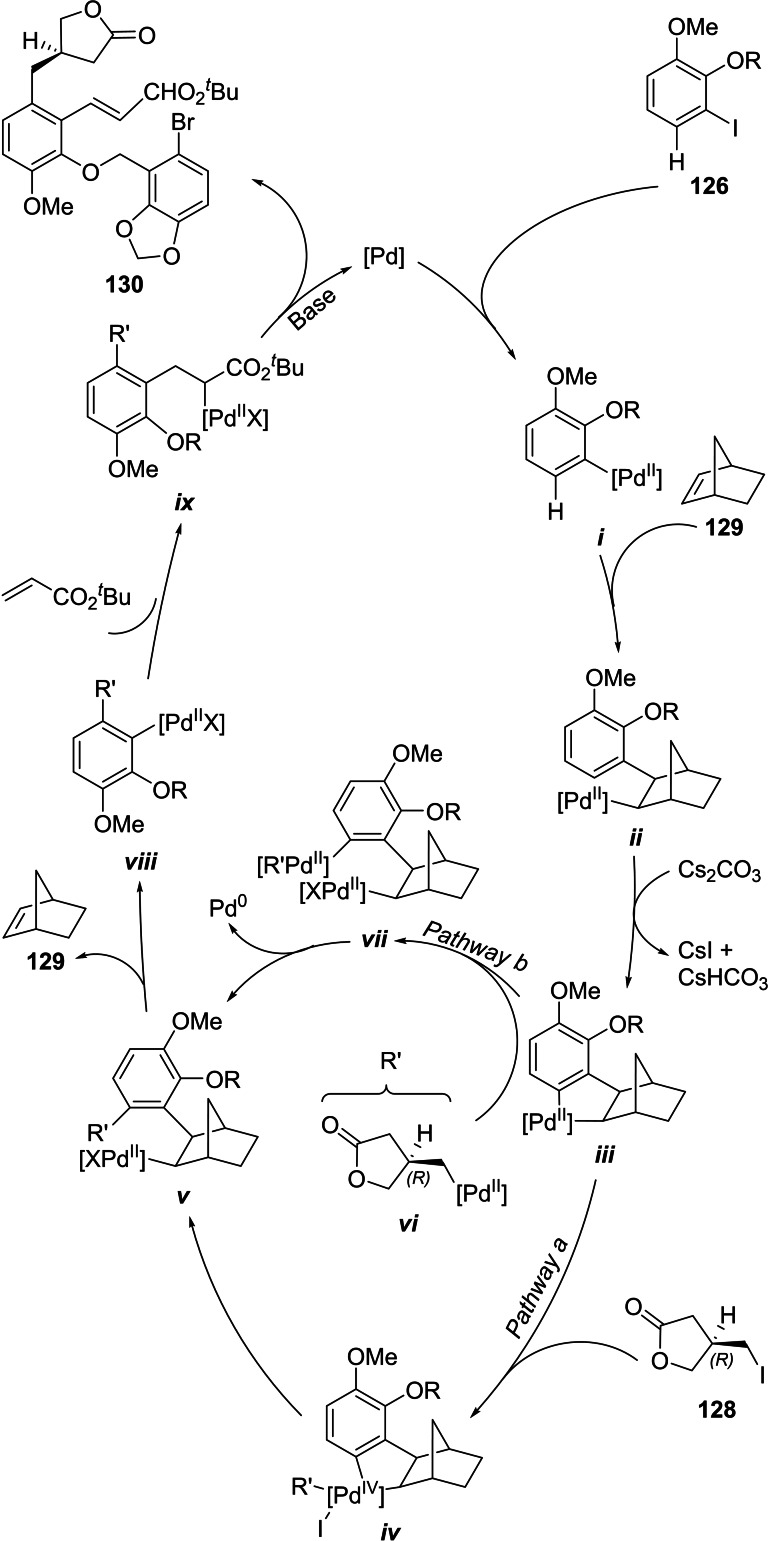
Proposed mechanism of the Catellani reaction for accessing **130**, a key intermediate for the total synthesis of linoxepin (**120**) and isolinoxepin (**132**).

This domino reaction seems to have an interesting mechanism, which is still not fully known. It has been proposed (Scheme 18) that the catalytic cycle is initiated by a Pd^0^ catalyst, which adds to the Ar−I bond of compound **126** to afford the palladium species ***i***.^[98]^ The latter then reacts with norbornene (**129**), which is used as a mediator that takes part in the cycle, facilitating the successive formation of the required C−C bonds between the starting iodoarene and the remaining pair of components of the process.

Carbopalladation of **129** gives intermediate ***ii***. The lack of a suitable β‐hydrogen in the structure of the bicycle prevents *syn* β‐hydride elimination, facilitating palladation of the position *ortho* to that of the halide in the starting material, yielding the palladacycle ***iii***, through base‐mediated C−H activation.

Therefore, the iodoalkane **128** is the first to be put in place, and this may follow two alternative pathways, including the oxidative addition to **128** to generate the Pd^IV^ intermediate ***iv*** (*Pathway a*), which upon reductive elimination forms intermediate ***v***.

Alternatively (*Pathway b*), generation of the Pd species ***vi*** and transmetallation between two different Pd^II^ centers, could afford intermediate ***vii*** and yield ***v*** after reductive elimination. Then, the retro‐carbopalladation of ***v*** would release **129** and furnish intermediate ***viii***, suitable to undergo attachment of the acrylate unit, by a mechanism reminiscent of the Mizoroki‐Heck reaction, completing the catalytic cycle and releasing the key intermediate **130**.

In view of the crowded nature of the double bond in the final product, an aldol reaction to intermediate **130** was performed by a Lemieux‐Johnson oxidative cleavage of the acrylate residue to afford an aldehyde, which was condensed with the lactone moiety under TiCl_4_ promotion, forming ring *E* in 98 % overall yield.

Next, the tetracycle **131** was subjected to a Mizoroki‐Heck reaction. When Et_3_N was employed as the base, under microwaves irradiation (130 °C) the reaction product was iso‐linoxepin (**132**), which could not be converted into the natural product, whereas the use of CsOAc under milder conditions (75 °C) gave linoxepin (**120**) in 76 % yield.

**Scheme 19:**
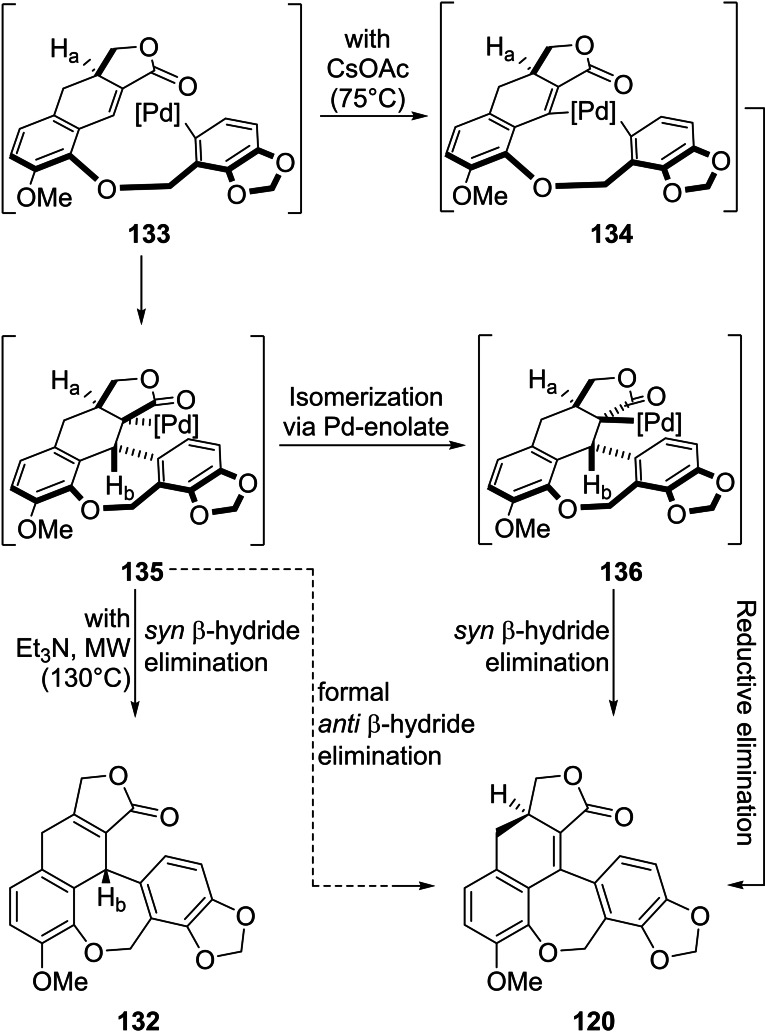
Proposed mechanism for the elaboration of the 7‐membered oxygen ring within the synthesis of linoxepin (**120**) and isolinoxepin (**132**).

The outcome of the reaction was rationalized as illustrated in Scheme 19. Under mild temperature conditions and with CsOAc as the base, the organopalladium species **133** can undergo a C−H activation by the highly electrophilic Ar–Pd species and afford the palladacycle **134**, which could further suffer a reductive elimination to give the natural product.

The latter could also result from intermediate palladium species **135**, through isomerization via a palladium enolate to yield **136** furthered by a *syn* β‐hydride elimination, providing an overall formal *anti* β‐hydride elimination path.

In the alternative pathway, under high temperature and with Et_3_N as the base, a *syn* β‐hydrogen elimination from the common intermediate **135** would afford isolinoxepin.

Interestingly, a combined Catellani/oxa‐Michael approach was used by Cheng *et al*. for their total synthesis of (−)‐berkelic acid.^[99]^ This complex spiroketal, which displays high cytotoxicity against the OVCAR‐3 ovarian cancer cell line, was isolated from an extremophilic *Penicillium* species.^[100]^

## 4 Conclusions

Natural products are usually complex and defiant polysubstituted structures, with different functionalizations, often characterized by the presence of one or more stereogenic centers. MCRs have given new impetus to the field of natural products by providing a whole new series of novel and efficient synthetic methodologies for their total synthesis.

Due to their ability to minimize the number of steps in the synthesis sequence while being tolerant to different functional groups, help to easily generate structurally complex compounds, and ‐as a consequence‐ simplify the synthetic process, they are rapidly gaining a relevant place among the handiest tools for the synthetic Organic Chemist.

This review summarizes the applications of different 3CRs to the synthesis of natural products. In most cases, these processes served as a platform to access critical parts of the proposed intermediates on the path toward the natural products. However, as a characteristic of natural product synthesis, often this effort also resulted in the development of new methodologies or strategies.

In addition, some sequences were possible in part thanks to significant methodological advances that have taken place in recent times, particularly those involving the use of new organometallic catalysts and the introduction of organocatalysis. However, the application of established photochemical, electrochemical, and enzymatic processes is still a methodological challenge, not only for the synthesis of natural products but also for their molecular diversification.

As a consequence of the development of shorter routes, an additional advantage of the use of 3CR approaches is the access to the target structures in quantities useful enough to inquire about their biological activity and possible biomedical applications.

Given the fact that our pharmacological arsenal is heavily grounded on or inspired by natural products, it is foreseeable that the use of MCR‐based strategies for their total synthesis (and the synthesis of related compounds) will also have an impact on different areas of biomedicine and allied disciplines, and that more MCR‐based syntheses should appear in the literature in the next future.

## Conflict of interests

The authors declare no conflict of interest.

5

## Biographical Information

*Teodoro S. Kaufman was born near Moisés Ville (Santa Fe, Argentina). He graduated in Biochemistry (1982) and Pharmacy (1985), and received his Ph.D. (1987, Prof. Edmundo A. Rúveda) from the National University of Rosario (UNR, Rosario, Argentina). After his postdoctoral training (University of Mississippi, USA), he returned to Rosario. Currently, Dr. Kaufman is a full Professor of the UNR and a Superior Researcher of the Argentine National Research Council (CONICET). He co‐authored over 185 papers and his scientific interests include the synthesis and evaluation of bioactive compounds (especially natural products) and their analogs, with emphasis on nitrogen and oxygen heterocycles*.

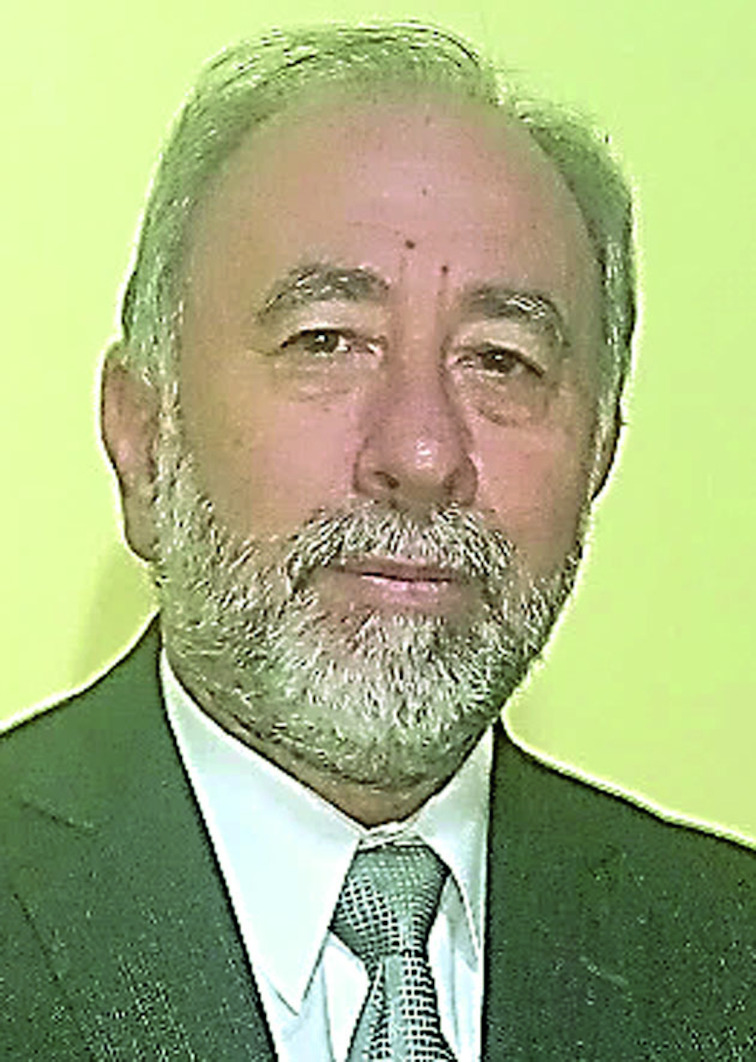

## Biographical Information

*Enrique L. Larghi was born in Rosario (Santa Fe, Argentina). He received his B.S. in Chemistry from the UNR (1997), while the M.Sc. degree (1999, Prof. Silveira) and Ph.D. in Chemistry (2003, Prof. Farias Morel) were awarded by the UFSM (RS, Brazil). After returning to Argentina and carrying out a fruitful experience in the pharmaceutical industry as Head of R&D, he joined Dr. Kaufman's group at IQUIR as a post‐doctoral fellow (2005). Currently, he is an Independent Researcher of CONICET and Adjunct Professor at UNR. His research interests focus on the synthesis of heterocyclic natural products and biological profile aspects*.

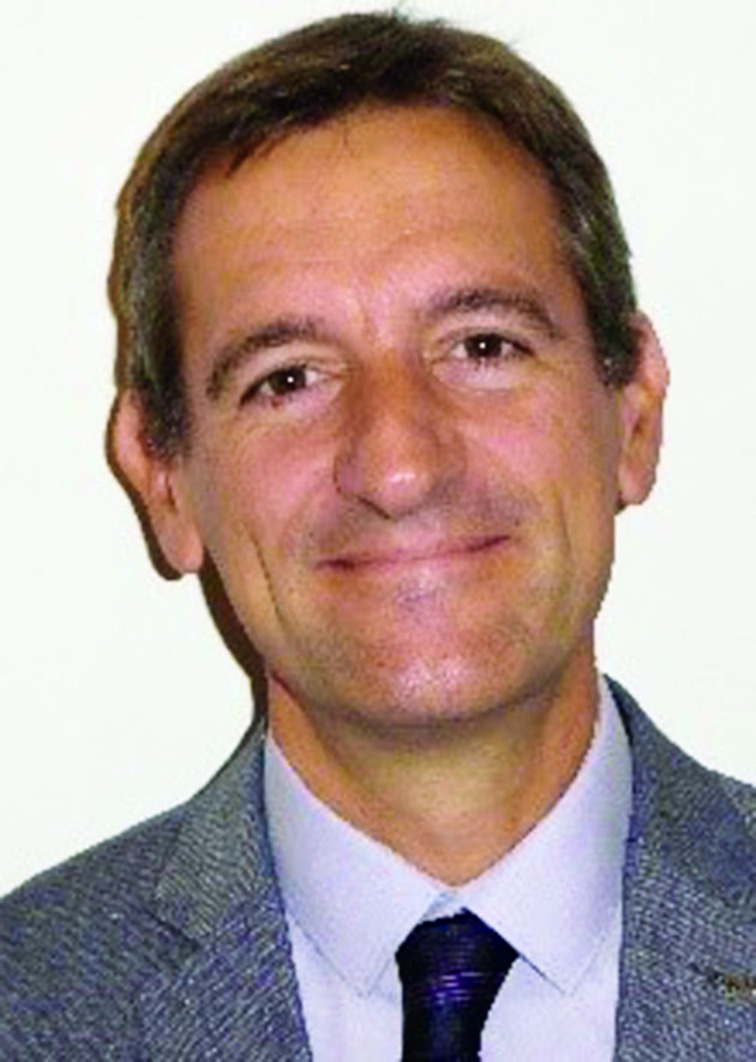

## Biographical Information

*Sebastián Simonetti was born in Chabás (Santa Fe, Argentina). He pursued his undergraduate studies at the National University of Rosario and obtained his PhD from the same university under the guidance of Dr. Teodoro S. Kaufman in total synthesis of natural products. Following a postdoctoral stay with Dr. Silvina Pellegrinet, funded by CONICET, and with Dr. Fabian Pfrengle, supported by the Alexander von Humboldt Foundation, he joined the Institute of Chemistry Rosario as a researcher in Prof. Kaufman's group. Currently, he is an Associate Researcher, focusing on the synthesis of glycosidic natural products and computational studies of organic reactions*.

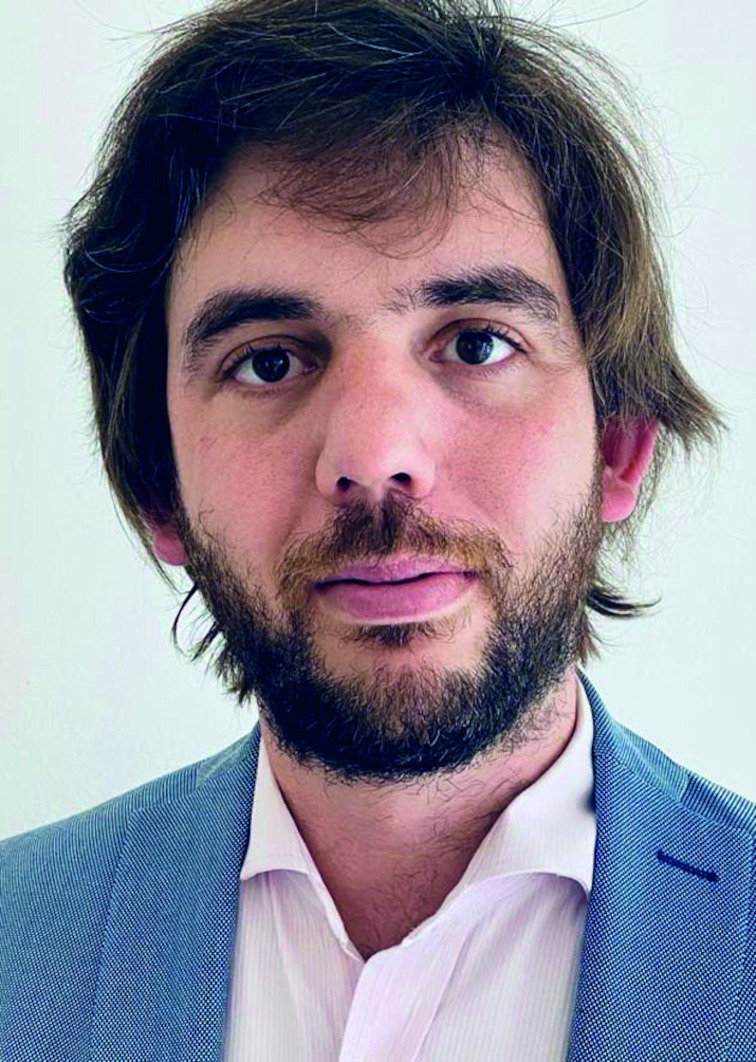

## Biographical Information

*Andrea B. J. Bracca was born in Rosario (Santa Fe, Argentina) and graduated in 2001 with a BS in Biotechnology from the National University of Rosario. She received her PhD in 2009 under the guidance of Prof. Kaufman. After a two‐year period of postdoctoral training, she returned to work in Dr. Kaufman's group as an Assistant Research Scientist at the Argentine National Research Council (CONICET) at the Institute of Chemistry of Rosario (IQUIR). Currently, Dr. Bracca is an Independent Researcher and develops research work in the area of the total synthesis of heterocyclic natural products and their most relevant analogs*.

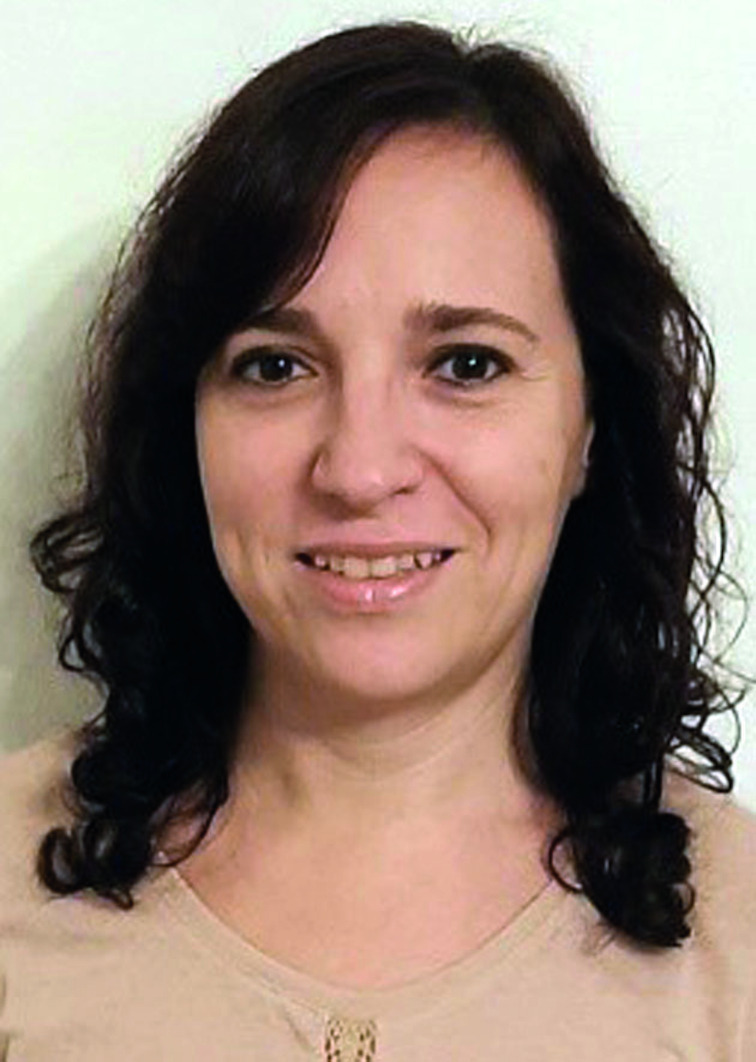

## Data Availability

Data sharing is not applicable to this article as no new data were created or analyzed in this study.
